# Exploring the relationship between urban green infrastructure connectivity, size and multifunctionality: a systematic review

**DOI:** 10.1007/s10980-025-02069-1

**Published:** 2025-03-10

**Authors:** Lei Li, Jeremy Carter

**Affiliations:** Department of Planning and Environmental Management, University of Manchester, Manchester, M13 9PL UK

**Keywords:** Green infrastructure, Multifunctionality, Connectivity, Ecosystem Services, Ecosystem Disservices, Urban Planning

## Abstract

**Context:**

Urban green infrastructure (GI) multifunctionality is widely valued within the academic literature, and underpins calls from policy makers to enhance and expand GI resources. However, there is a gap in understanding concerning how GI connectivity and size influence GI multifunctionality outcomes.

**Objectives:**

The objectives are to: (1) present the current status of research on urban GI multifunctionality (encompassing ecosystem services and disservices) and the GI traits of connectivity and size; (2) identify relationships between these topics within the literature; (3) provide research insights and present actionable GI planning recommendations based on the findings of the research.

**Methods:**

A systematic review of 139 academic sources (2010–2023) was conducted following the PRISMA guidelines.

**Results:**

Key findings include that multifunctionality themes are more commonly considered within research exploring GI connectivity across urban boundaries than within them, where a wider range of flows of ecosystem functions and associated services (and disservices) are enabled. Also, research predominantly focuses on multiple large GI sites, with limited attention to the multifunctionality of single small GI sites that are commonly found in dense urban areas.

**Conclusions:**

Greater consideration is needed of how the manipulation of GI size and connectivity influence multifunctionality outcomes, whilst also recognising the threat of ecosystem disservices emerging as a result of such actions. Through uncovering gaps in understanding concerning these issues, and highlighting topics benefiting from stronger research foundations, this research can support GI policy, practice and research in realising GI multifunctionality outcomes in urban settings, whilst minimising ecosystem disservices.

**Supplementary Information:**

The online version contains supplementary material available at 10.1007/s10980-025-02069-1.

## Introduction

Green infrastructure (hereafter GI) incorporates ecosystems and natural processes into the fabric of cities and urban landscapes, in turn offering multiple benefits linked to responding to challenges posed by climate change and rapid urbanization. In addition to GI, numerous complementary and interrelated concepts focus on achieving these overarching objectives. They include nature-based solutions (NBS), and ecosystem management, protection and restoration approaches (Kabisch et al. 2016; Li et al. 2021).

Structurally, GI embraces various forms from living walls and green roofs on buildings to larger scale wetlands, forests and natural habitat networks (UK Green Building Council 2015). In the UK, Natural England (2009, 2021) clarifies that GI consists of green spaces and other environmental features situated within and surrounding the built environment, and delivers benefits across spatial scales from local urban sites, through the urban fringe and into adjoining rural areas. In the US, the Water Improvement Act of 2019 positions GI as an approach that utilises natural processes to manage stormwater flows and reduce negative impacts from urban runoff on water quality and quantity metrics. The European Commission (2019, p. 1) extends the scope of functions provided by GI, highlighting that it can support the delivery of multiple ecosystem services (hereafter ES), defining GI as “*a strategically planned network of natural and semi-natural areas with other environmental features designed and managed to deliver a wide range of* *ecosystem services”*. GI can therefore be positioned as a multi-scalar and multifunctional approach that can be utilised by urban policy makers, planners and practitioners to create more holistic and spatially integrated strategies for sustainable urban development.

Multifunctionality is fundamental to understanding and framing urban GI. This topic has been discussed conceptually from 1999, with notions of multifunctional green spaces considered within academic research since 2005 (Wang and Banzhaf 2018). GI multifunctionality has been extensively explored in the literature (Lovell and Taylor 2013; Liquete et al. 2015; Connop et al. 2016; Kim and Song 2019; Wang et al. 2021; leBrasseur 2022a; Korkou et al. 2023), with previous studies demonstrating GI benefits associated with hydrological, ecological, recreational and economic perspectives (Hassan et al. 2005). Broadly, GI can help to tackle urban environmental impacts, protect and promote ecosystem functions, improve human well-being and strengthen local economies (Brzoska and Spage 2020; Felappi et al. 2020; Li et al. 2020).

This paper focuses on themes and approaches linked to the enhancement of urban GI multifunctionality, with particular attention paid to the size of, and connectivity between, GI sites in this respect. The connectivity between physically separate ecosystem elements in non-urban settings has been explored to understand implications for dispersal processes (e.g. the movement of organisms, species, and resources between disconnected ecosystems), and how connectivity from this perspective influences the extent of ES and ecosystem disservices (hereafter EDS) (Chatzimentor et al. 2020). According to the theories of metapopulation ecology, the size and connectedness of habitat patches are identified as important factors influencing metapopulation dynamics in natural environments (Hanski 1994a, b). Research within natural environments also demonstrates that species abundance and diversity is influences by the size of habitat patches (Ovaskainen 2002; Lindenmayer et al. 2010, 2015). However, levels of understanding and awareness of the relationships between ecological connectivity and ecosystem multifunctionality in urban settings is more limited (LaPoint et al. 2015; Simion et al. 2023). There are exceptions, however. For example, a variety of well-known ecological theories have been applied to urban environments, such as niche theory and its many applications (e.g. related to species distribution models, trait-based analyses and studies of the temporal or spatial partitioning of resources within an ecological community) (Parris 2018). Such studies have identified factors influencing biodiversity and ecosystem multifunctionality characteristics in urban areas, including the heterogeneity of land uses (which generates a variety of habitat types and niches), the greater influence of non-native species and humans, fragmentation of habitats, poor linkages between GI and grey infrastructure and high edge to core ratios of GI patches (ibid).

Whilst there is abundant knowledge concerning factors influencing the capacity of biodiversity to thrive in urban environments (Sweeney et al. 2007; Niemelä, 2011), a clear understanding of how urban GI characteristics, which can also be referred to as GI traits, influence multifunctionality outcomes is lacking. For example, the lack of attention paid to small GI sites within current research (Mesimaki et al. 2019; Vega and Küffer 2021; Egerer et al. 2024) limits opportunities for enhancing GI connectivity in dense urban areas, and realising their cumulative beneficial impacts and potential contributions to urban sustainability. This is particularly evident when looking beyond studies focused on biodiversity and ecosystem multifunctionality towards a wider range of environmental, social and economic ES themes. However, relevant conceptual studies are emerging including Andersson et al.’s (2021) social-ecological traits framework which can be utilised to better comprehend social-ecological patterns, interactions, dynamics, and tipping points within and across complex urban systems, thereby informing governance and decision making for urban sustainability. The size of urban GI sites is recognised as an important determinant of the extent of multifunctional ES outcomes (Valente et al. 2020), with research emerging on the impact GI size on species characteristics (Stott et al. 2015; Ibanez-Alamo et al. 2020; Vega and Küffer 2021) and ES linked to human health and wellbeing (Soga et al. 2014; Stott et al. 2015).

This study aims to contribute to this agenda by presenting a systematic literature review focused on the status of research (published between 2010 and 2023) that addresses the relationships between urban GI multifunctionality (encompassing ES and disservices) and the GI traits of connectivity and size. It represents the first such review to focus on these topics in an urban context, with the outcomes targeted at supporting policy makers, practitioners and researchers in their efforts to optimise the multifunctionality of urban GI. The three research objectives guiding this study were: (1) to explore the current status of research on urban GI multifunctionality (encompassing ES and disservices) and the GI traits of connectivity and size; (2) to identify relationships between these topics within the literature; (3) to develop research insights and provide actionable GI planning recommendations based on the findings of the research.

### **Literature review**

Multifunctionality concerns the potential for GI to benefit people and ecosystems through the provision of associated social, economic and ecological functions (Hansen and Pauleit 2014; Korkou et al. 2023). There is a wealth of evidence establishing the multiple environmental, social-cultural and economic benefits that GI conservation, enhancement and expansion has the potential to bring to urban areas (Mell 2010; Meerow and Newell 2017; Pauleit et al. 2017; Engström et al. 2018; Chatzimentor et al. 2020; Felappi et al. 2020; Li et al. 2020; O’Donnell et al. 2021; Di Pirro et al. 2022; Korkou et al. 2023). Research has also identified and classified different urban ES, with connections made to specific GI forms (e.g. parks, green roofs etc.) that have the potential to support the realisation of these services in urban areas (Gómez-Baggethun and Barton 2013; Brzoska and Spage 2020).

It is also increasingly recognised that urban GI can generate negative impacts. Lyytimäki and Sipilä (2009) were the first to propose the concept of EDS linked to urban GI. They stressed the importance of assessing this topic, warning that overlooking nuisances and losses stemming from urban ecosystem functions and neglecting related EDS could negatively impact the perception and management of urban green spaces. Despite this, negative ecosystem functions and disservices have received limited attention within GI research relative to ecosystem services (Choi et al. 2021; Guo et al. 2022). There are exceptions (e.g. Lyytimäki and Sipilä, 2009; Gómez-Baggethun and Barton 2013; Cariñanos et al. 2017; Liu et al. 2018; Wamsler et al. 2020; Choi et al. 2021; Guo et al. 2022; Thapa et al. 2023), with EDS identified linked to themes including the presence of pest species, conflict and competition between species living within urban habitats, and risk to humans from dangerous or poisonous animals including exposure to diseases, pathogens and their vectors (Gómez-Baggethun and Barton 2013; Liu et al. 2018). Studies have also highlighted public concerns linked to associations between urban GI and animals, insects, toxic plants, aesthetics and crime, in addition to risks related to water bodies (Williams et al. 2019; Roman et al. 2021; Plieninger et al. 2022; Liu et al. 2024).

There is also emerging consideration of trade-offs between ES and EDS. Zhang and MacKenzie (2024) revealed that relationships between ES and EDS vary significantly and are strongly context-dependent, shaped by the attributes of GI components, biophysical conditions, and the perspectives of local stakeholders. For example, Amorim et al. (2021) observed that urban parks with high aesthetic and recreational value are more likely to trigger green gentrification, further accelerating social stratification. Further, better connectivity between green spaces can facilitate biodiversity enhancement in urban areas, yet may in turn negatively impact individuals with pollen allergies and enhance the risk of vectors (e.g. rats and ticks) transmitting infectious diseases to humans (Lõhmus and Balbus 2015; Heylen et al. 2019; Hansford et al. 2023). Similarly, bodies of water and wetlands are important for urban climate adaptation and mitigation but can also generate negative effects including serving as habitats for mosquitoes and toxic algal blooms.

Multifunctionality features prominently within good practice GI planning principles identified within the academic literature. Pauleit et al. (2017) highlighted four such principles, namely multifunctionality, connectivity, green-grey infrastructure integration and socially inclusive planning. Monteiro et al. (2022) identified the same four principles, adding the need for multi-scalar approaches, diversity, applicability, governance and continuity to this list. Huang (2023) positioned multifunctionality, connectivity and integration as core GI planning principles, also citing diversity, multi-scalar approaches, adaptability, sustainability, digitalization and public participation as co-principles. Multifunctionality is clearly a foundational GI planning principle, driven by the desire to utilise often limited amounts of urban greenspace as effectively as possible. Designing urban GI to enhance its ability to offer multiple ecosystem functions and services simultaneously is therefore an important consideration (Cook et al. 2024). Here, the traits of GI connectivity and size/scale are related supporting the realisation of multifunctional outcomes in practice (Hansen and Pauleit 2014; Huang 2023; Cook et al. 2024).

It is common to conceptualise GI as a network of interconnected blue and green spaces extending within and beyond the administrative boundaries of cities and urban areas (Santo-Tomás Muro et al. 2020). GI connectivity can be separated into structural and functional dimensions (Hansen and Pauleit 2014; Hou et al. 2023). Structural connectivity concerns the extent to which GI elements are interconnected through physical links based on the spatial structure of the landscape (e.g. via eco-corridors and greenways), whereas functional connectivity relates to the capacity of the landscape to, for example, facilitate the movement of species, water and humans (Brooks 2003; Hou et al. 2023). Within urban areas, research on GI connectivity is generally structurally oriented (Hou et al. 2023). Functional connectivity is more commonly considered in the context of ecological networks at landscape scales or in non-urban settings (Chatzimentor et al. 2020), although LaPoint et al. (2015) found that research specifically on ecological connectivity in urban areas often had a functional foundation.

In principle, urban GI multifunctionality can be enhanced by creating networks of interconnected green spaces. Indeed, Monteiro et al. (2020, 2022) emphasise the need to create well-connected green space networks to promote the multiple benefits that GI has the potential to offer. Further, the EU GI Strategy directly links GI multifunctionality to ecological connectivity (Chatzimentor et al. 2020). Similarly, a lack or loss of connectivity can in turn decrease the capacity of GI to provide ES such as pollination, species migration and breeding, and seed dispersal (Estreguil et al. 2019). Looking beyond the movement of species and their genetic material, enhancing GI connectivity can also encourage other benefits including pollutant dispersal and cool air transfer (Reuters and Kapp 2021). However, it is important to recognise that enhancing GI connectivity may also intensify EDS, with clear links evident between GI connectivity, species and their movement, and associated human exposure diseases. For example, this can occur through processes including the expansion of breeding grounds for disease-spreading mosquitoes and increasing human-tick-deer interaction and the corresponding risk of transmitting Lyme disease to humans (Hansford et al. 2023).

In addition to connectivity, themes linked to the size of GI sites and the extent to which urban GI resources are multi-scalar in nature appear across lists of GI planning principles (Pauleit et al. 2017; Monteiro et al. 2022; Huang 2023). The SLOSS (single large or several small) debate, which originated within ecology and conservation biology disciplines during the 1970s and 1980s, has focused on determining whether a single large or several small nature reserves are more effective for species conservation (Ovaskainen 2002; Lindenmayer et al. 2010, 2015; Tjørve 2010). Here, Tjørve (2010) argues that the best strategy will depend on the geographic location and species being considered.

The SLOSS debate provides a useful framework for considering the size trait from the perspective of urban GI, and appears to be extending into urban and GI planning research. Related studies largely remain conceptual and theoretical rather than empirical. Valente et al. (2020) have suggested applying SLOSS concepts to explore whether fewer large nature reserves/urban parks or several small green areas are more effective in promoting urban ES and enhancing human-wellbeing. Reflecting its origins in ecology and conservation biology, urban research into themes linked to the size of GI patches is particularly focused around habitats and species. For example, Egerer et al. (2024) argued that in addition to large urban green spaces, dispersed small green spaces can also foster a diverse range of both managed and spontaneous taxonomic and functional diversity among plants and animals through the provision of resources such as native vegetation as a shelter, shrubs for food, and flowering trees. Considering wildflower species richness, Vega and Küffer (2021, p. 1) found that small patches of urban green space are “…*punching above their weight*…”, and that connecting small urban green space patches together and to larger green spaces could further enhance their potential as wildflower habitats through enabling natural colonization and strengthening existing populations.

longside the SLOSS framework, the notion of ‘land sharing’ versus ‘land sparing’ offers a similar scalar approach to conceptualising, researching and managing the relationship between different urban land uses and the provision of ES (Lin and Fuller 2013). This approach also explicitly addresses the provision of green space (Stott et al. 2015). Land sharing relates to maintaining multiple smaller greenspaces (e.g. parks, gardens) within a lower density built environment, whereas land sparing concerns maintaining separate, large and contiguous green spaces within densely built up urban areas (Stott et al. 2015). This approach links to the more habitat-focused SLOSS framework, with connections evident between the single large/land sparing, and several small/land sharing concepts. Different urban ES outcomes have been associated with land sharing and land sparing strategies. For example, land sparing is important for delivering higher invertebrate and avian species numbers in urban areas (Soga et al. 2014; Stott et al. 2015; Ibanez-Alamo et al. 2020). However, a land sharing strategy can help to ensure that humans benefit from urban green spaces and the associated ES that they can offer (Soga et al. 2014; Stott et al. 2015). Ultimately, therefore, a mix of GI scales and size configurations is needed to capture the multiple ES that urban GI can offer.

The GI traits of connectivity and size have an important bearing on the multifunctionality of urban GI. Individually, these themes have received attention within the academic literature, especially concerning multifunctionality and connectivity, and here focus has particularly been on ES linked to habitats and species. Emerging research linked to GI size themes also has an urban ecology focus. From a broader perspective, the status of existing research into urban GI multifunctionality and relationships to the GI traits of connectivity and size is unclear. Further, whilst GI multifunctionality is widely discussed in the literature, there is limited recognition in the literature of the trade-offs and potential EDS linked to maximizing GI’s multiple functions, for example through enhancing the connectivity of GI sites. A better understanding of the extent and focus of current research on these topics is needed to address this gap, to inform the direction of future research enquiry, and to support practical approaches to increase the multifunctionality of urban green spaces. To achieve this, our novel literature review comprehensively assessed ES and EDS and maps their interrelationships to GI traits (specifically connectivity and size) and GI forms.

## Systematic literature review methodology

**Fig. 1:**
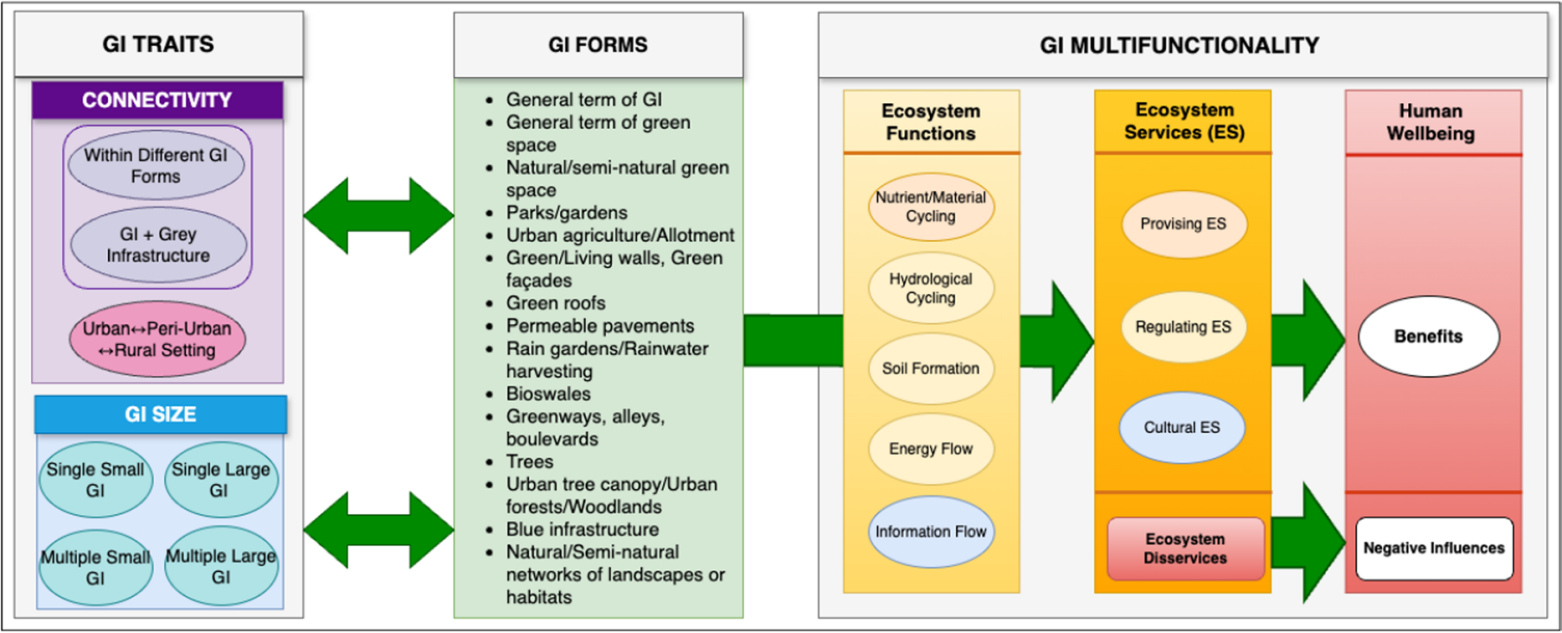
Conceptual framework linking GI traits, forms and multifunctionality themes (adapted from Millennium Ecosystem Assessment 2005; Hansen and Pauleit 2014; Douglas 2017; United States Environmental Protection Agency 2023)

Building on the outcomes of the literature review outlined above, a conceptual framework was developed to guide the systematic literature review method and to structure the analysis and discussion of its findings. This framework is presented in Fig. 1. It brings together the themes underpinning the systematic literature review, the central focus of which is to clarify how current academic research is exploring the traits of GI connectivity and size in the context of understanding and delivering urban GI multifunctionality. The GI forms around which this research is based were also identified, with the USEPA (2023) classification of GI forms adapted to provide a framework for this element of the study.

The conceptual framework highlights that two dimensions of GI connectivity were explored within the systematic literature review, both linking to the notion of structural connectivity which is the dominant approach found within urban GI research (Chatzimentor et al. 2020; Hou et al. 2023). Firstly, structural (or physical) connections between different GI forms and between GI and grey infrastructure were considered. ‘Within different GI forms’ relates to green-green, blue-green or blue-blue infrastructure connectivity (i.e. between the GI forms listed in Fig. 1). ‘Between GI and grey infrastructure’ relates to connectivity between GI forms (listed in Fig. 1) and grey infrastructure. Grey infrastructure refers to engineered systems and structures that support urban life and the provision of essential services (e.g. buildings, transport infrastructure, energy infrastructure, water supply and waste water treatment infrastructure and flood defenses) (Dong et al. 2017; Masseroni et al. 2018; O’Donnell et al. 2021; Thapa et al. 2023). Connectivity is also considered between GI sites within urban settings, and across urban boundaries into peri-urban and rural areas. The SLOSS framework guides the conceptualization of the GI size trait, with research studies evaluated according to whether they consider single small, single large, multiple small, and multiple large GI sites. GI size is evaluated based on consideration of GI form. Within this study, small GI includes green/living walls, green roofs, permeable pavement, bioswales, and trees. The remaining GI forms included in Fig. 1 are considered large GI (aside from the general terms).

Finally, the conceptual framework incorporates GI multifunctionality. Academic researchers are increasingly utilising ES frameworks to classify GI functions and services, structure GI multifunctionality analyses, and interpret the interrelationships between natural and social capital. Prominent approaches include the Millennium Ecosystem Assessment’s four types of ES (supporting, provisioning, regulating and cultural), the Common International Classification of Ecosystem Services (CICES V5.1) and the classification of EDS (Gómez-Baggethun and Barton 2013; European Commission 2016; Brzoska and Spage 2020; Plieninger et al. 2022). For example, Hansen and Pauleit (2014) proposed a conceptual framework to assess GI multifunctionality from a social-ecological perspective, building on the cascade model (Haines-Young and Potschin 2012) that sequences the transition from ecosystem functions to ES and finally to human well-being benefits. Our conceptual framework utilises the cascade model, adapted from Hansen and Pauleit (2014), which links ecosystem functions, ecosystem services and impacts on human wellbeing. The Millennium Ecosystem Assessment’s ES classification provide the basis for this element of the study. The conceptual framework also acknowledges that urban GI can generate EDS that negatively impact human wellbeing.

### Search strategy

#### Data collection and identification

A keyword search was conducted to identify potential sources to include in the systematic literature review. There was no limitation made on the date of publication, with the search output identifying sources published from 2010 to the middle of November 2023 (when the search was undertaken). The final searching string was formatted for use in Web of Science (WOS) and Scopus, focusing on titles, abstracts, keywords. The search terms for ‘GI, multifunctionality, connectivity, size and planning’ were linked with the Boolean operator “AND”, while the Boolean operator “OR” was used to link synonyms and related terms. The final search string is provided below:

((TITLE-ABS-KEY(“Green infrastructure*” OR “Blue-green infrastructure*” OR “Green space*”)) AND (TITLE-ABS-KEY((Urban*) OR (City*))) AND (TITLE-ABS-KEY(“Multifunction*” OR “Multiple functions*”)) AND TITLE-ABS-KEY(“Connect*” OR “Connection*” OR “Connectivity*” OR “Network*” OR “Size*” OR “Scale*”)) AND (TITLE-ABS-KEY(Planning*)) AND (LIMIT-TO (LAN-GUAGE, “English”)).

This study only focused on English language publications. Whilst this may exclude valuable regional studies published in other languages, this approach ensured the systematic review remained methodologically sound (i.e. by ensuring standardisation and consistency concerning terminology used within the literature) and practically manageable for the research team accounting for their language skills and expertise.

#### Data selection process

Sources identified within WOS and Scopus, and all associated data included the full-text and initial citation report, title, authors, sources title, publication year, total and average citations, and citations per year, were uploaded into the EPPI-reviewer software programme. This software programme was used to manage and analyse the source data. Duplicates were removed automatically following data upload. Full-text publications not available in Google Scholar or through institutional library subscriptions were requested from corresponding authors via ResearchGate.

**Fig. 2:**
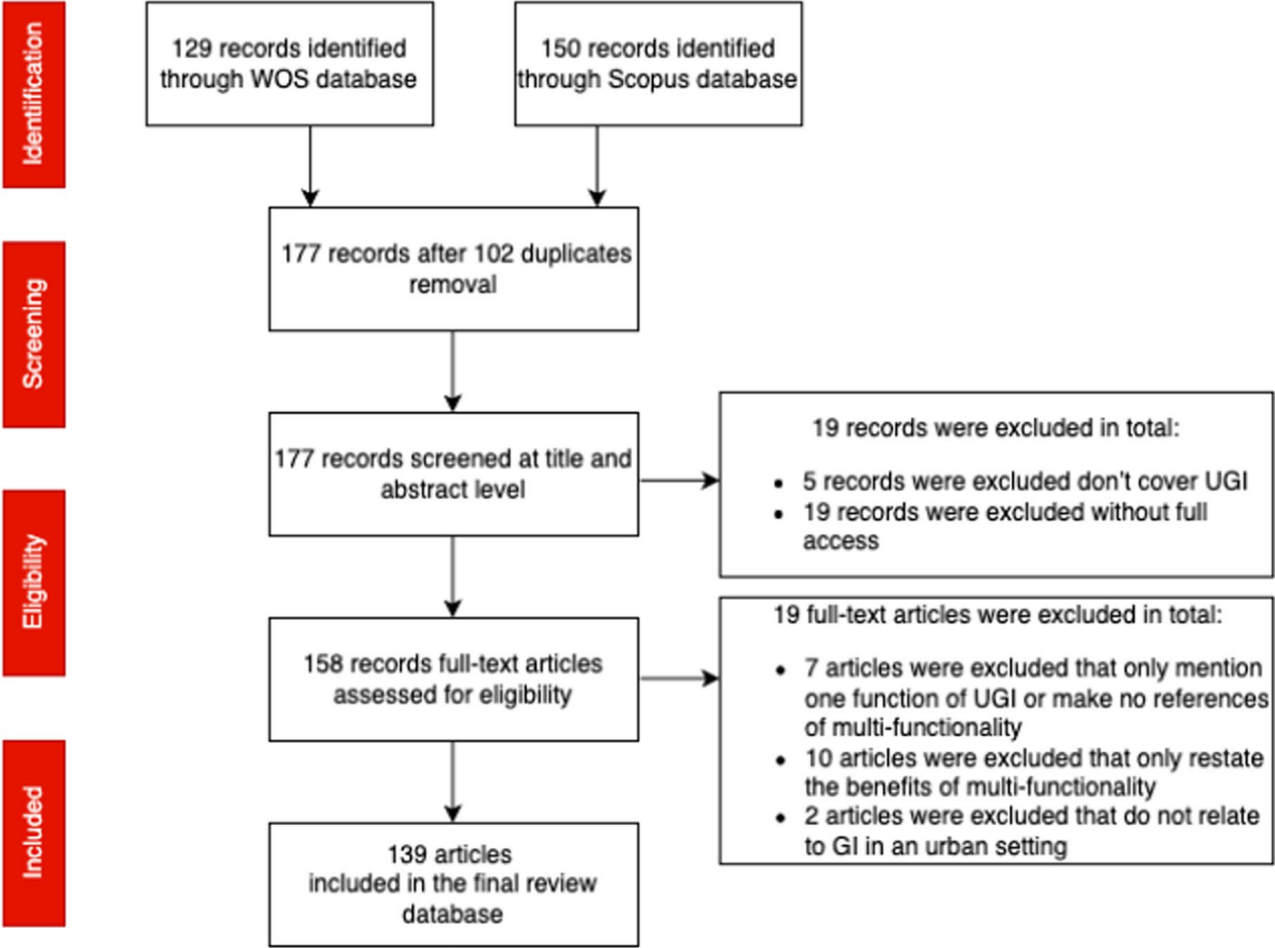
PRISMA flowchart showing the process to identify review sources

The process of searching for and analysing literature is depicted using a PRISMA flowchart (Moher et al. 2009) (Fig. 2). The screening process was undertaken at two levels, title and abstract and then full text. The inclusion and exclusion criteria used to screen potential sources are provided in Table 1. 38 publications were excluded in total, 19 at each level of screening. Two reviewers checked the consistency of screening process to reach agreement on the final list of sources to include in the database. Following screening, 139 sources were identified for review.

**Table 1:** Review inclusion and exclusion criteria

Inclusion criteria	Exclusion criteria
Eligible population: Global urban GI (UGI) studies Eligible intervention: Studies analysing urban GI from the aspects of concept/theory, practice/implementation, planning/policy Eligible outcome: Studies exploring urban GI multifunctionality, with a focus on urban GI connectivity and size traits	Criteria applied at title and abstract stage: Exclude publications that do not cover urban GI Exclude publications not written in English Exclude publications without full access Criteria applied at full-text stage: Exclude publications that only mention one function of urban GI and make no reference to multifunctionality Exclude publications that restate the benefits of multifunctionality without presenting a conceptual or empirical research contribution Exclude publications that do not relate to GI in an urban setting (but include GI studies that explore urban-peri-rural connectivity)

### Coding process

Data from the 139 publications selected to include within the review was extracted and coded using EPPI-Reviewer. Coding descriptions, questions and options are listed in Appendix 1. Definitions of each code can be found in Appendix 2. The codes were developed around the conceptual framework (Fig. 1) and covered GI forms, size, connectivity and multifunctionality. Descriptive data on the publications, covering countries of origin, methodology and study scales was also gathered. The common international classification of ecosystem services (CICES V5.1) and related research studies on ecosystem services and disservices were used to develop sub-codes for the multifunctionality theme (adapting Gómez-Baggethun and Barton 2013; European Commission 2016; Brzoska and Spage 2020; Plieninger et al. 2022) (see Appendix 3). In our review, supporting ES are not included within the ES codes. It is rare for supporting ES to be provided by urban GI, and previous urban GI research has not assessed this ES classification (Gómez-Baggethun and Barton 2013; Brzoska and Spage 2020).

To ensure consistency at the coding stage, two reviewers checked and discussed emerging coding issues to reach consensus on the review outcomes. Firstly, two reviewers collaboratively developed a coding framework based on the conceptual framework and research objectives. The coding framework was pilot-tested on a small subset of papers to identify ambiguities or discrepancies, which were resolved through discussion and refinement of the framework. The first reviewer independently coded the assigned papers and the second reviewer cross-checked the coding of papers for data validity and verification. Two reviewers met regularly to discuss coding progress, resolve disagreements and ensure consistent application of the coding framework. For papers deemed particularly complex, both reviewers independently coded them, and results were compared to ensure agreement. After the coding process, a final review of the coded data was conducted independently to identify and address any inconsistencies before analysis.

### Analysis process

The analysis of the reviewed sources took place within EPPI-Reviewer. This included developing an overview of the characteristics of the sample of publications and undertaking a detailed analysis of the core themes sitting at the heart of this study and the relationships between these themes. Following the conceptual framework (Fig. 1), the relationships between these traits, GI multifunctionality and GI forms were analysed and visualised using Sankey diagrams. Associations between the GI traits of connectivity and size and the geographic focus, methodology and study scale of related literature sources were also analysed.

## Results

### Overview of the publications included in the review

**Fig. 3:**
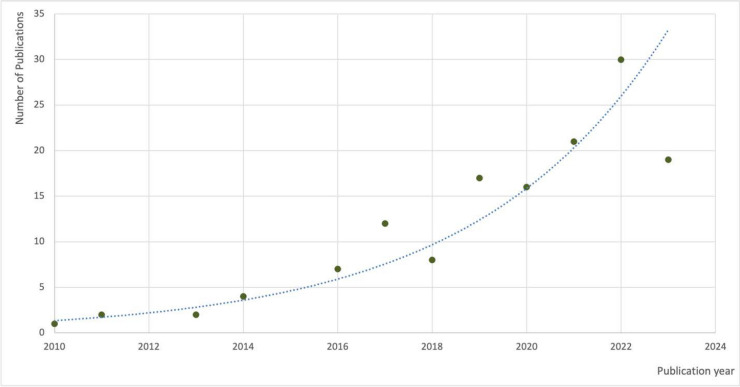
The number of publications meeting the review inclusion criteria (2010–2023) (The literature search finished in November 2023, and therefore the figure for 2023 does not represent a full calendar year)

Publications exploring urban GI multifunctionality are a relatively recent occurrence, with the first source fitting the inclusion criteria for this review published in 2010. Since then, aside from minor reductions in 2017 and 2020, there has been an upwards trajectory in annual publication numbers peaking in 2022 with 30 articles (see Fig. 3). Our literature search ended in November 2023, accounting for the lower number of sources identified within 2023.

**Fig. 4:**
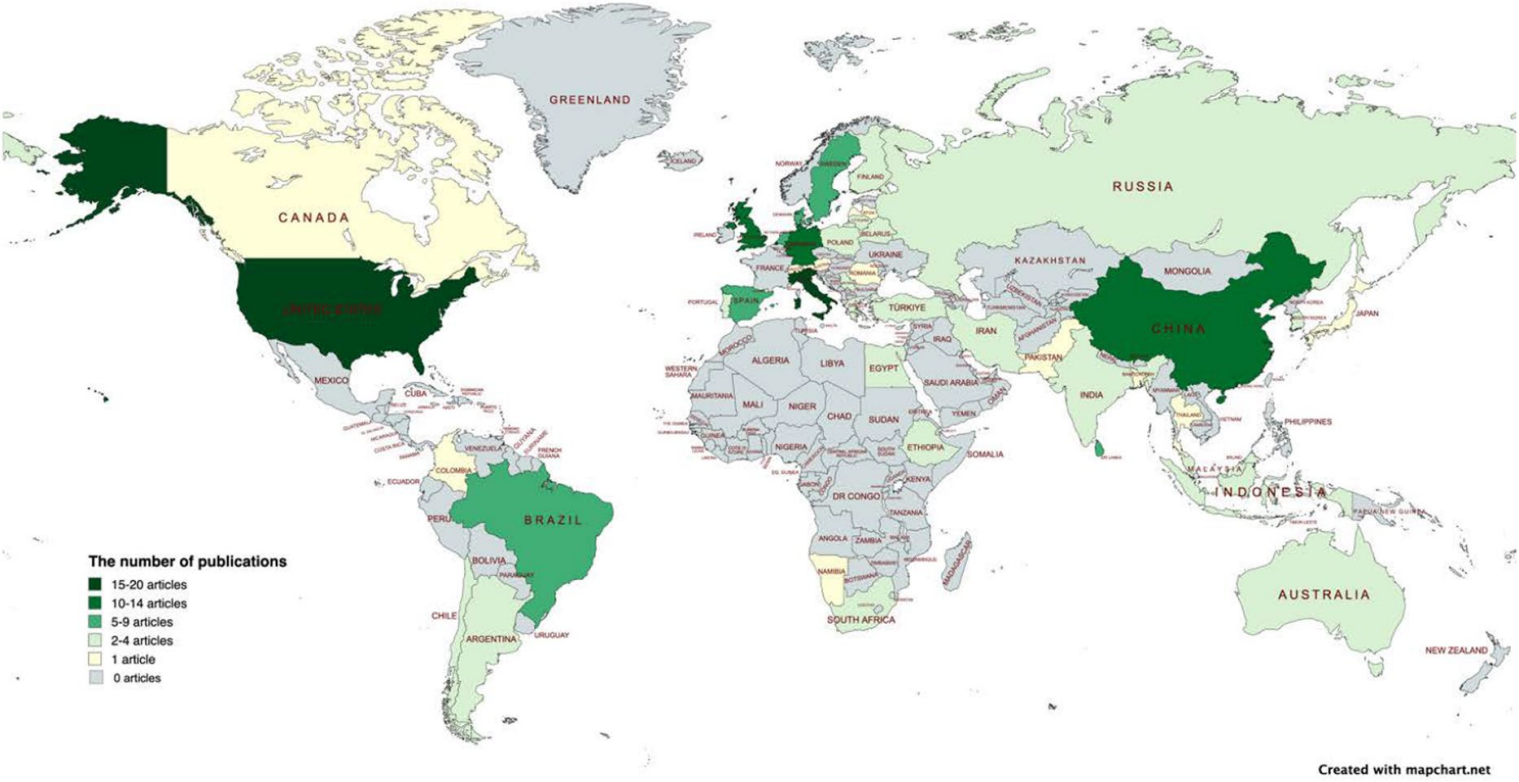
Geographical classification detailing the number of publications including research meeting the systematic literature review inclusion criteria

Figure 4 presents 5 groups of countries classified according to the number of publications referring to these countries. 45% of the publications focused on GI multifunctionality in a European context. This reflects Europe’s leading role in GI policy and practice, with European Commission supporting research projects and practical case studies in this field over recent decades. Asia and North America are also the focus of a considerable amount of research in this field, representing 18% and 13% of the publications respectively. Publications presenting research originating from Africa, South America, and Oceania remain relatively rare. The top 11 countries, ranked by the number of publications included in the review database, were the US (19 publications), Italy (16 publications), Germany (14 publications), UK (12 publications), China (11 publications), Spain (8 publications), Brazil (6 publications), Denmark, Sweden, Netherlands and Sri Lanka (5 publications each).

Methodologically, empirical studies were most common (114 publications), followed by modelling (50 publications), review (42 publications) and conceptual (22 publications) approaches (see Appendix 2 for definitions of each methodological approach). 55 studies employed one type of methodology, whilst 79 studies employed two types of methodology. Only four studies incorporated three types of methodology. The most common approach was to combine empirical and modelling methods (49 publications), followed by studies that incorporated empirical and review approaches (23 publications). Case studies were often included within the reviewed publications. Indeed, 85 sources included a single case study, with a further 39 referring to multiple case studies.

**Fig. 5:**
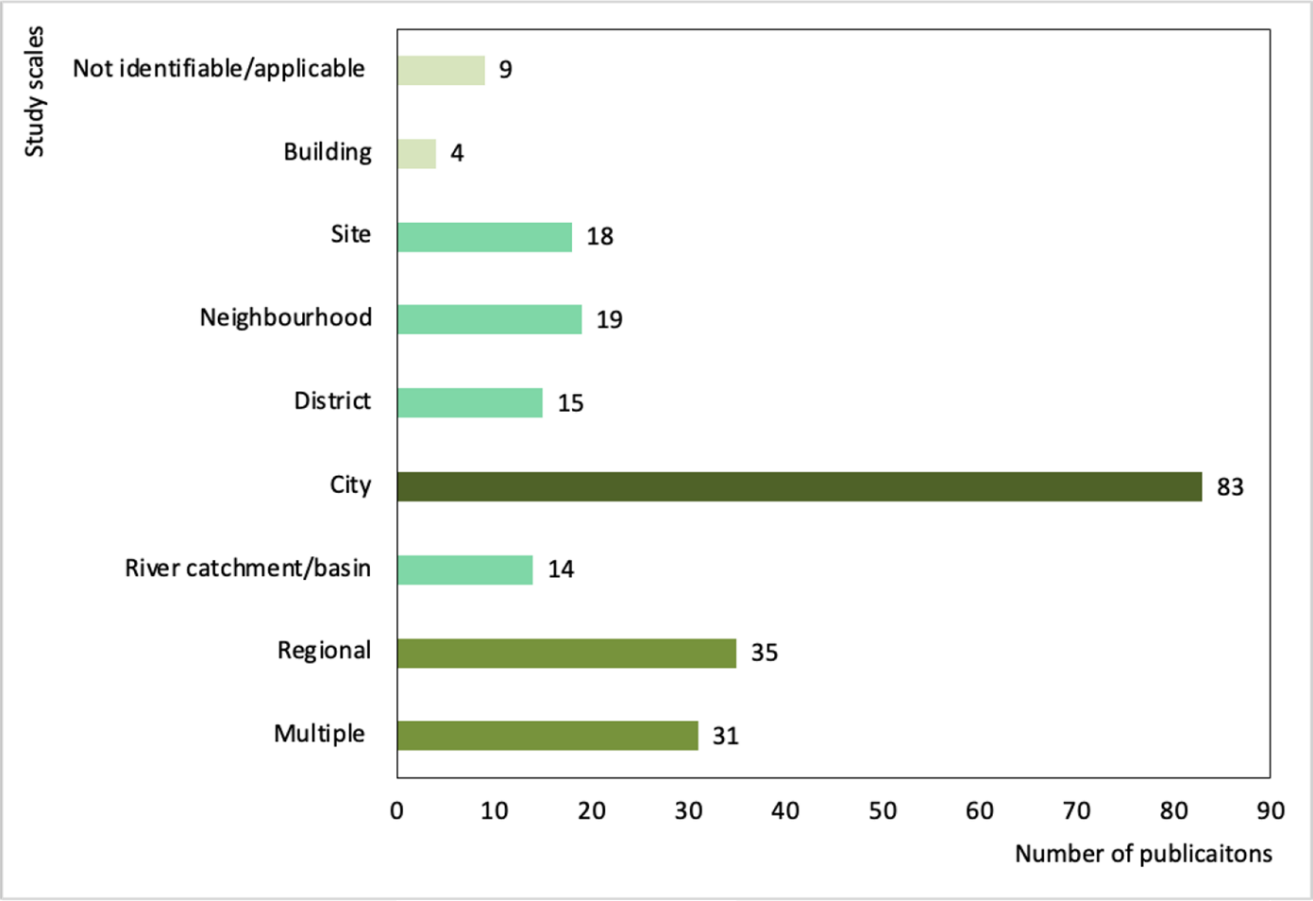
Spatial scales considered by the publications included within this review

GI multifunctionality research is undertaken at a range of different spatial scales (Appendix 2 defines the 9 scales considered within this review). Figure 5 clarifies that research into GI multifunctionality at the city scale is most common, followed by regional scale research. Studies at smaller spatial scales including districts, neighbourhoods and GI sites (e.g. green roofs) are less common. GI multifunctionality studies undertaken at the building scale are rare. Generally, GI multifunctionality studies focus on a single spatial scale. Within this review, 99 publications (71% of the total sample) explored one spatial scale, with 19 publications (14%) considering two scales. The remainder considered more than two spatial scales.

### Relationships between GI multifunctionality and the traits of GI size and connectivity

#### Overview of research on GI multifunctionality

Looking across the sample of 139 publications, regulating ES (126 publications) and cultural ES (123 publications) were most commonly referred to, followed by provisioning ES (61 publications) and finally EDS (24 publications). ‘Social relations and recreation’ was the most frequently cited individual ES (102 publications), followed by ‘human well-being improvement’, ‘biodiversity conservation’ and ‘water flow regulation and runoff mitigation (including flood control)’, cited within 86, 80 and 77 publications respectively. The most commonly cited EDS were ‘green gentrification’ (10 publications), closely followed by ‘dangerous or poisonous animals’ (9 publications).

**Fig. 6:**
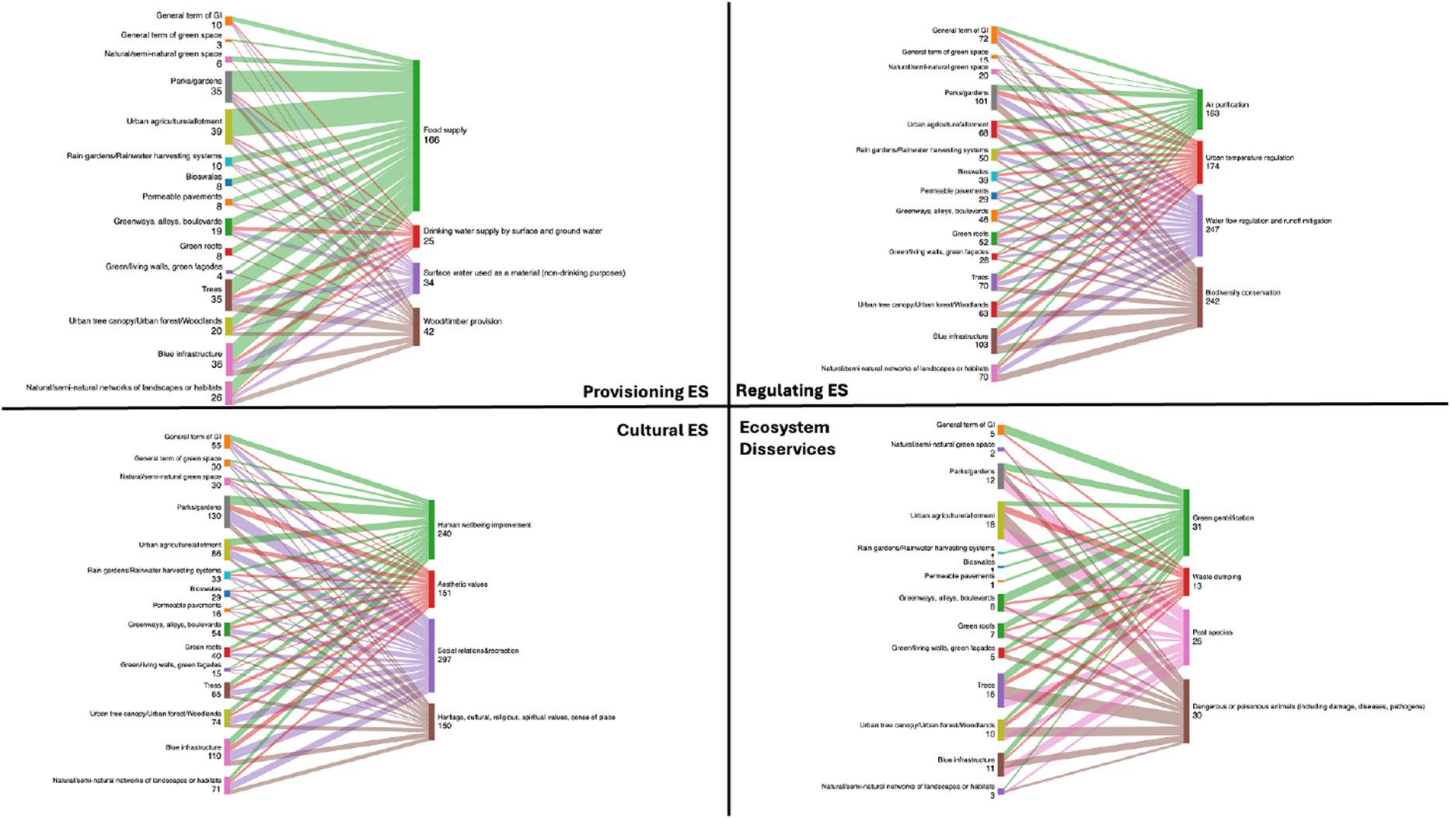
Sankey diagrams visualising the relationship between GI forms and the provision of individual ES and EDS (numbers indicate the frequency of references to the topic within the review sample of publications)

The review also identified links between individual ES and disservices and the GI forms they are associated with. The full results for 25 ES and 15 EDS are presented using a heatmap in Appendix 4, with Fig. 6 displaying and comparing the results for the top four individual ES within the provisioning, regulating and cultural categories, in addition to the top four EDS. The extent of links between GI forms and specific ES and EDS, as identified within the literature, are represented line thickness of the lines in Fig. 6, with the number of links found within the literature also provided (also see Appendix 4). Essentially, this visualization provides a rich picture of how dominant ES and EDS types are associated with different GI forms within the literature. Figure 6 (and Appendix 4) can be interrogated to identify relationships between a wide range of specific ES, EDS and GI forms. For example, it can be seen that food supply is the most common provisioning ES, and is particularly associated with urban agriculture and parks and gardens. Figure 6 also establishes that urban agriculture contributes the most provisioning ES, with 39 links identified, with food supply being the most commonly specific ES.

Regarding cultural ES, Fig. 6 clarifies that ‘social relations and recreation’ was particularly connected to parks/gardens (49 publications), as was ‘improving human well-being’ (35 publications), with the majority of these studies (63%) linked to European countries. From the perspective of regulating ES, ‘biodiversity conservation’ was commonly associated with parks/gardens (34 publications), blue infrastructure (31 publications), and natural/semi-natural networks of landscapes or habitats (26 publications). Further, ‘water flow regulation and runoff mitigation (including flood control)’ was strongly linked to blue infrastructure (35 publications) and parks/gardens (28 publications). Amongst the 35 publications linking ‘water flow regulation and runoff mitigation’ to blue infrastructure, 51% highlighted connectivity across urban/peri-urban/rural boundaries. Examples include the development of blue infrastructure networks guided by the new ‘water town’ urbanism concept in China (Zhang et al. 2022), enhancing landscape connectivity through multifunctional GI corridor modelling and design in the US (Zhang et al*.* 2019b), and designing stormwater runoff and water quality initiatives within large watershed-scale community developments in the US (Yang and Li 2013).

Intuitive results were observed for provisioning ES. ‘Food supply’ was especially connected to urban agriculture/allotment (30 publications). ‘Surface water’ (mainly used as a material and not as drinking water) was most closely linked to blue infrastructure (7 publications). Regarding food supply, relevant examples from Sri Lanka include using home gardens as sustainable urban agroforestry to promote household well-being (Jayakody and Basu 2022) and utilising multiple small GI sites including home gardens, community gardens and privately owned lands for food production (Dona et al. 2022).

Finally, Fig. 6 establishes that EDS were particularly related to parks/gardens (12 publications), trees (10 publications) and urban agriculture/allotment (9 publications). ‘Pest species’ and ‘dangerous or poisonous animals (including damage, diseases, pathogens)’ display the closest relation with urban agriculture/allotment, followed by parks/gardens and trees. Specific related examples about ‘dangerous/poisonous animals’ and ‘pest species’ emerged in home gardens in Sri Lanka (Jayakody and Basu 2022), during the governance of UGI in informal settlements of Namibia (Wijesinghe and Thorn 2021), and the evaluation of seaweed farming as an eco-engineering strategy for ‘blue’ shoreline infrastructure in Singapore (Heery et al. 2020).

#### Overview of research on GI size

This study considered the extent to which published academic literature considers GI size in the context of delivering multifunctional GI outcomes. Drawing on the SLOSS framework, it was established that studies focusing on multiple large GI sites were dominant (65 publications), followed by those exploring multiple small GI sites (22). Research focused on single large GI sites was rare (3 publications), whilst no studies considered a single small GI site from a multifunctionality perspective. Multifunctionality research is therefore primarily associated with multiple GI sites. Specific examples of studies focused on multiple large GI sites include those exploring ecological-corridors for species migration and genetic exchange in Italy (Cannas et al. 2018), enhancing the multifunctionality of peri urban landscapes in Madrid’s suburbs (Fernández-Pablos et al. 2021), how peri-urban farmlands can support sustainable urban development in Europe (Rolf 2021) and Argentina (Baldini et al. 2022), the role of integrating watercourses to re-establish Tehran’s identity as a green city (Chamanara and Kazemeini 2016), and the extension of Blue-Green infrastructure networks in Australia to address losses in natural capital and ES (Ghofrani et al. 2020). Publications focused multiple small GI sites include those covering topics including how home gardens can enhance household wellbeing in Sri Lanka (Jayakody and Basu 2022), the delivery of multiple ES via trees in India (Thapa et al. 2023), the impact of green roofs on energy efficiency and local biodiversity conservation in Italy (Astiaso Garcia et al. 2014), and the contribution of nature-based stormwater management approaches (including rain gardens, permeable pavement and green roofs) to sustainable city transitions in Sweden (Zischg et al. 2019). In addition, certain studies compare different sizes of GI sites from a social-economic perspective, in terms of public preferences and perceptions concerning the usage of green space (Lahoti et al. 2023), and the impact of the size and extent of GI on property prices (Czembrowski and Kronenberg 2016).

**Fig. 7:**
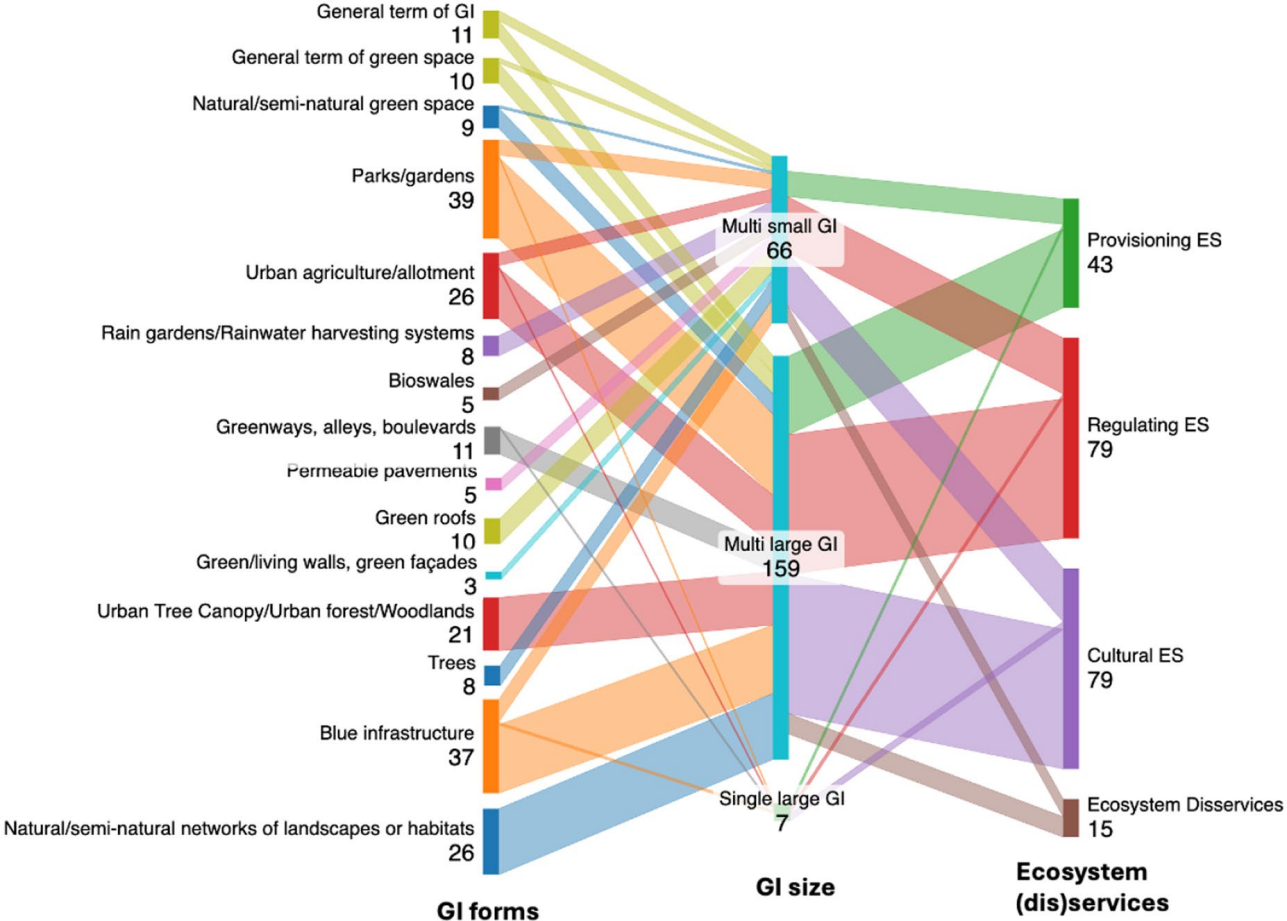
Sankey diagram visualising the relationship between GI form, size and ecosystem services/disservices themes (numbers indicate the frequency of references to the topic within the review sample of publications)

Figure 7 visualises relationship between GI size, urban ES and EDS, and different GI forms. Where studies focused on multiple large GI sites, parks/gardens (32 publications), blue infrastructure (27 publications), natural/semi-natural networks of landscapes or habitats (26 publications), urban tree canopy/urban forests/woodlands (21 publications), and urban agriculture/allotment (20 publications) were the key GI forms considered. Where research focused on multiple small GI sites, green roofs (10 publications), blue infrastructure (9 publications), rain gardens/rainwater harvesting systems (8 publications), and trees (8 publications) were the dominant associated GI forms.

Connections to ES and disservices were also explored in the context of the GI size trait. Here, it was established that publications discussing provisioning, regulating and cultural ES were particularly associated with multiple large GI sites, although this does reflect the dominance of studies focusing on this GI size category. Figure 7 confirms that regulating and cultural ES were primarily associated with the exploration of multiple large GI sites. Provisioning ES were considered less frequently, and again related studies tended to be associated with research on multiple large GI sites. Regarding EDS, clear relationships to the GI size trait were less apparent, highlighting that EDS arise across GI sites of varying sizes. Indeed, 8 papers identifying EDS were associated with multiple large GI sites (three mentioned pest species, and 3 referred to dangerous or poisonous animals), with 7 papers discussing EDS in the context of multiple small GI sites (5 mentioned dangerous or poisonous animals, 3 addressed pest species, and another 3 discussed fear and stress).

#### Overview of research on GI connectivity

##### GI connectivity within/across urban boundaries

**Fig. 8:**
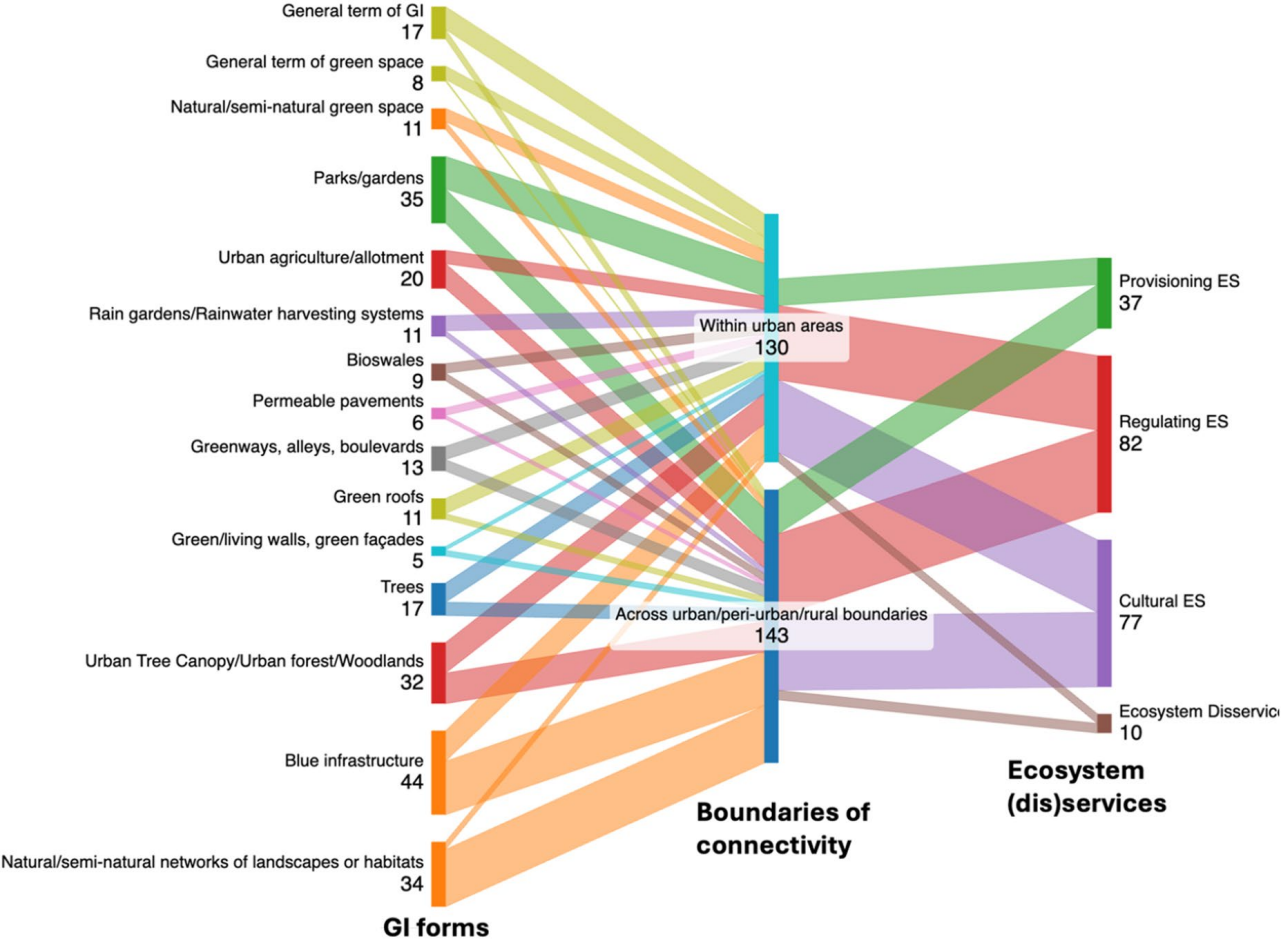
Sankey diagram visualising the relationship between connectivity within and across urban boundaries, GI form and ES and disservices GI provided by GI (numbers indicate the frequency of references to the topic within the review sample of publications)

Regarding boundaries of connectivity (Fig. 8), studies exploring this trait ‘across urban/peri-urban/rural boundaries’ were slightly more common (46 publications) than studies exploring connectivity ‘within urban areas’ (42 publications). Figure 8 highlights that the dominant GI forms explored within studies on GI connectivity within urban areas were parks/gardens (17 publications), blue infrastructure (16 publications), and urban tree canopy/urban forests/woodlands (16 publications). Where publications explored connectivity across urban/peri-urban/rural boundaries, natural/semi-natural networks of landscapes and habitats (30 publications), blue infrastructure (28 publications), and parks/gardens (18 publications) often provided the foundation for related research activity. Examples of research focused on natural/semi-natural networks of landscapes and habitats that extend across urban-peri-rural boundaries include studies on ecological corridors (Cannas et al. 2018), green belts (Verdú-Vázquez et al. 2017; Kirby and Scott 2023) and GI networks (Carranza Orantes et al. 2017; Ahmed et al. 2019; Verdú-Vázquez et al. 2021; Zhang et al. 2022; Hou et al. 2023).

Publications focused on connectivity across urban/peri-urban/rural boundaries demonstrated a stronger association to provisioning (23 vs. 14 publications) and regulating ES (43 vs. 39 publications) than those investigating connectivity within urban areas. For example, studies linked to agricultural and horticultural activities are predominantly concentrated in peri-urban areas, offering ES including food production and ecological conservation (Olsson et al. 2016; Rodenbiker 2020; Baldini et al. 2022; Dona et al. 2022).

The systematic literature review also analysed relationships between GI forms, connectivity and ES and disservices. Each provisioning ES included in this review featured more prominently in studies focusing on GI connectivity across urban/peri-urban/rural boundaries than those considering GI connectivity within urban areas. Similarly, regarding cultural ES, ‘education and research’ and ‘heritage, cultural, religious, spiritual values, sense of place’ have higher numbers of publications linking to connectivity across urban/peri-urban/rural areas than those addressing connectivity within urban areas. This same pattern is observed for regulating ES including soil quality, water purification, biodiversity conservation, disease and pest control. Concerning EDS, the number of papers with connectivity ‘within urban areas’ and ‘across boundaries’ was the same (5 papers each). Key findings include that green gentrification featured more prominently within studies exploring GI connectivity within urban areas (3 papers), followed by allergies (2 papers), whilst risk linked to dangerous or poisonous animals (including damage, diseases, pathogens) (3 papers) and pest species (2 papers) were associated with studies considering GI connectivity across urban/peri-urban/rural areas. Therefore, it is apparent that where GI connectivity operates across urban rather than within urban boundaries, there is the potential for flows of a wider range of ecosystem functions and associated services and disservices to be enabled.

##### Connectivity between forms of infrastructure

**Fig. 9:**
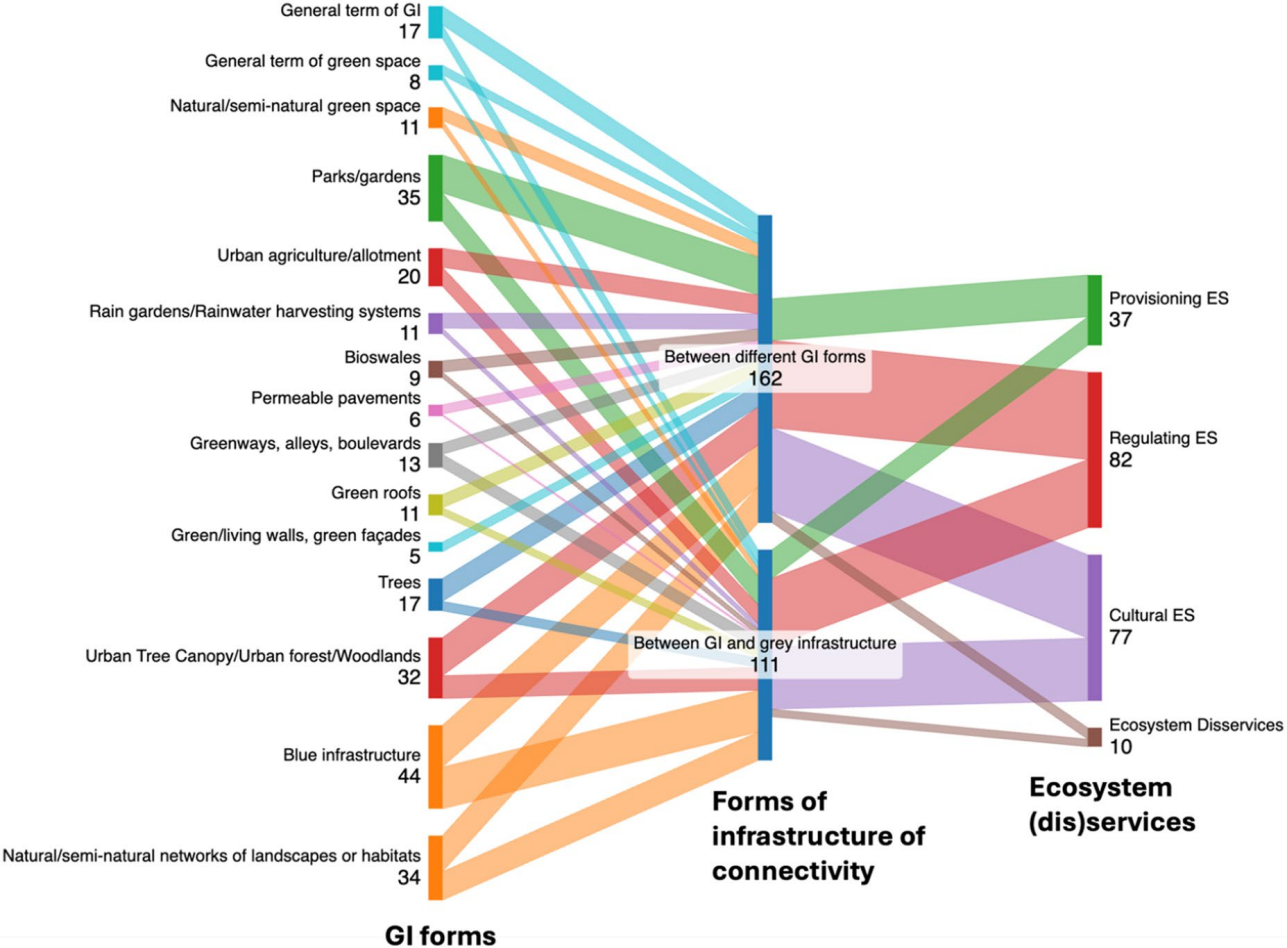
The Sankey diagram presents the relationship between GI form and forms of infrastructure of connectivity and Ecosystem (dis)services that GI provided (numbers indicate the frequency of references to the topic within the review sample of publications)

Figure 9 demonstrates that studies exploring connectivity ‘between different GI forms’ were more common (49 publications) than those considering this trait from the perspective of connections ‘between GI and grey infrastructure’ (39 publications). Connectivity between different GI forms particularly encompassed research on blue infrastructure (22 publications), parks/gardens (20 publications), and urban tree canopy/urban forests/woodlands (20 publications). Where studies considered connectivity between GI and grey infrastructure, blue infrastructure (22 publications), parks/gardens (15 publications), and natural/semi-natural networks of landscapes or habitats (15 publications) were the dominant GI forms.

Publications considering different forms of infrastructure connectivity commonly addressed three broad themes: water management, biodiversity conservation and urban planning and design. Regarding water management, connections are evident between blue infrastructure or blue-green infrastructure (BGI) and grey infrastructure such as canals, reservoirs and drainage systems, with the aim of managing water quality and quantity issues (Ahmed et al. 2019; Yang and Li 2013; Huang 2023; Sheng et al. 2023). Other examples include eco-engineering approaches to plant seaweed into urban seawalls to generate multifunctional blue shorelines (Heery et al. 2020). Infrastructure connectivity is also targeted at biodiversity conservation and education through, for example, urban rewilding (Zefferman et al. 2018), and supporting bird migration by connecting sandbanks with artificial wetlands (Kim et al. 2018). Concerning urban planning and design, research concentrates on the integration of BGI/GI with built environment features including pavements and buildings to improve landscape connectivity and consequently enhance ES provision (Zhang et al*.* 2019a; Ghofrani et al. 2020; Lourenço et al. 2020; leBrasseur 2022a; Thapa et al. 2023). Further studies of this type link to sustainable urban planning and design goals including water-town design (Zhang et al. 2022), eco-architecture (Astiaso Garcia et al. 2014), urban planning for compact cities (Hansen et al*.*, 2019) and arid zones (Mahmoud and Selman 2010, 2011), new forms of urban agriculture (La Rosa et al. 2014), urban landscape revitalization (Song 2023), and smart cities (Turaga et al. 2020).

Publications focused on connectivity between different GI forms (6 papers) have slightly more discussions of EDS than those where connectivity between GI and grey infrastructure is being considered (4 papers). More specifically, green gentrification was more prominently featured in studies examining GI connectivity between different GI forms (4 papers), while risks associated with dangerous or poisonous animals (3 papers) and pest species (3 papers) were more commonly linked to studies exploring GI connectivity with grey infrastructure.

## Discussion

Existing literature emphasises the value of GI multifunctionality (Li et al. 2020; Korkou et al. 2023), and identifies the various functions and benefits that GI can provide (Liquete et al. 2015; Wang and Banzhaf 2018; Wang et al. 2021; Chen et al. 2022; leBrasseur 2022a, b). Our study highlights that academic reviews reach different conclusions regarding the prominence or absence of particular GI functions and benefits. For example, in contrast to Wang and Banzhaf (2018), who noted that cultural ES are not widely considered within GI literature, our review identified that cultural ES (especially linked to human wellbeing, social relations and recreation) feature strongly across the literature. Further, Kim and Song’s (2019) review identified economic themes (e.g. increased property values and the creation of green jobs) as the most referenced GI benefit, whereas our study identified that the literature features numerous ES more prominently than economic benefits. The reality appears to be that academic research perceives and studies GI multifunctionality differently, influenced by factors including methodologies employed and the geographical origin and disciplinary focus of researchers. Given the variability of urban areas, acknowledging and accepting divergence in perceptions of GI multifunctionality provides space for researchers to move further and to explore themes including barriers and enablers to achieving GI multifunctionality in practice.

In addition to identifying the incidence of ES within the literature, our systematic review goes further and analyses the relationships between ES (and EDS) linked to urban GI and the corresponding GI forms that deliver them (also see, Kim and Song 2019; Choi et al. 2021; Cook et al. 2024). Broadly, our findings confirm a tendency in the literature to associate engineered GI forms with water-related ES, whilst more natural/semi-natural GI forms are more often linked with themes including biodiversity conservation and human well-being improvement (see Cook et al. 2024). However, our review is distinguished from other studies by its coverage of a wider range of GI functions and the incorporation of 15 types of ecosystem disservice, which are not commonly considered within the academic literature. Figure 6 and Appendix 4 capture the key findings of this review on the association between GI services and GI forms, providing a useful reference point for researchers and practitioners looking to better understand these relationships to inform GI planning, action and research. Here, it is important to acknowledge that evaluating an excessive number of GI functions and forms can face planners and decision makers with a paradox of choice, and that the systematic evaluation of a smaller range of specific ES that suit local contexts and targeting collaboratively agreed objectives for urban GI therefore has merit (Cook et al. 2024).

This systematic literature review adds to and extends our understanding of urban GI multifunctionality. Indeed, the primary goal of our review was not only to identify ES linked to urban GI and the GI forms associated with their delivery, which has been the subject of recent studies (e.g. Korkou et al. 2023), but to use insights gained on these topics as a platform to investigate the extent to which academic research considers the traits of GI connectivity and size in the context of realising GI multifunctionality. This represents a novel contribution that has not been addressed systematically to date. This paper also responds to broader gaps in understanding concerning urban GI connectivity and multifunctionality and the associated provision of ES through GI spatial planning (e.g. identified by Pozoukidou 2020; Stoia et al. 2020; Chen et al. 2022), and related to EDS. These particular contributions are now discussed in more detail.

### *Exploring existing literature linking GI connectivity and GI multifunctionality*

Firstly, considering connectivity between different infrastructure forms, our review identified more references to connectivity ‘between different GI forms’ than ‘between GI and grey infrastructure’ (see Fig. 9). In many countries, GI is particularly directed towards stormwater management. Indeed, GI is defined by the USEPA as a sustainable stormwater management approach, with LID in the US, WSUD in Australia, SUDS in the UK, and China’s Sponge City Program all based around utilising GI for managing water quality and quantity issues. Consequently, concerning connectivity between different GI forms, there is abundant research based around the integration of BGI approaches (e.g. Fletcher et al. 2015; Eckart et al. 2017; Kuller et al. 2019; Deely et al. 2020; Donnell et al. 2020; Everetta et al. 2021; Simões et al. 2022). Looking beyond water, existing literature also considers connectivity between urban GI forms including parks, gardens, urban forests and tree canopies, and the delivery of ES including climate change adaptation, reducing heat stress, improving air quality, enhancing biodiversity and improving human health (Bowler et al. 2010; Giannakis et al. 2016; Vieira et al. 2018; Nastran et al. 2019; Zhang et al. 2019a; Egerer et al. 2024). Building on these findings, further studies are needed on themes including which urban GI forms can be effectively connected, processes and governance arrangements for facilitating connectivity between GI forms, and the multifunctionality outcomes that emerge from such activities as a result.

The integration of green and grey infrastructure is identified as an important consideration for GI planners and designers (Pauleit et al. 2017; Huang 2023). This systematic literature review established that blue infrastructure, followed by parks/gardens and natural/semi-natural networks of landscapes or habitats, are key GI forms being explored from the perspective of connecting to grey infrastructure (see Fig. 9). Grey infrastructure forms that GI is commonly connected to include sewers and drains, retention and detention basins, levees and floodwalls, reservoirs and dams, commercial and residential buildings, transport systems (e.g. cycle paths, roads and highways), public spaces and plazas (Hansen et al. 2017). Stormwater management was the prominent target ES where green-grey infrastructure connectivity is explored within the literature (Drosou et al. 2019; Chen et al. 2021; Huang 2023), with implementation of related approaches evident in countries including Indonesia (Drosou et al. 2019) and China (Chan et al. 2018; Leng et al. 2020) linked to urban flood management initiatives (Chen et al. 2021). This review also identified that green-grey infrastructure connectivity is associated with achieving biodiversity conservation and sustainable urban design goals (e.g. Mahmoud and Selman 2010, 2011; Astiaso Garcia et al. 2014; Kim et al. 2018; Zefferman et al. 2018; Hansen et al. 2019; Zhang et al. 2022). In addition, research from American cities (McPhillips and Matsler 2018; Bell et al. 2019) revealed economic motivations to advocating green-grey infrastructure connectivity to increase the cost-effectiveness of infrastructure measure and to open up opportunities for obtaining GI funding through demonstrating synergies and co-benefits to other forms of infrastructure. Similarly, promoting green-grey infrastructure connectivity may help to avoid conflicts and perceptions of competition between different types of infrastructure when looking to access funding (Zimmerman et al. 2019). Building on these insights, more research is needed to explore how multifunctionality outcomes vary where different types of green-grey infrastructure connectivity are achieved, and how GI and urban and GI planners and practitioners can work collaboratively with infrastructure providers to optimize this dimension of urban GI connectivity.

Concerning spatial boundaries, GI multifunctionality is marginally more likely to be considered within literature focused on GI connectivity operating across urban/peri-urban/rural boundaries rather than within urban areas (see Fig. 8). GI connectivity across urban boundaries and into their hinterlands, facilitated by natural/semi-natural networks of landscapes (e.g. eco-corridors and green belts), enables a broader range of flows of ecosystem functions and associated services to be realised. This chimes with the importance afforded to green space networks in European countries (Kurdoğlu and Kurt 2017; Cannas et al. 2018; Díaz-Varela et al. 2021; Verdú-Vázquez et al. 2021; Bajić et al. 2022; Isola et al. 2022; Santiago-Ramos and Hurtado-Rodríguez 2022) and green belts in the UK (Amati and Taylor 2010; Thomas and Littlewood 2010; Kirby and Scott 2023) where cultivating cross-boundary connectivity feature strongly as motivating factors. However, governance of cross-boundary GI networks, for example related to utilising landscape scale GI approaches to support Natural Flood Management, presents significant challenges due to the mismatch between administrative and biophysical boundaries (Carter et al. 2018, 2024). More specifically, fragmented decision-making processes, diverging policy objectives, resources disparity, land ownership and management issues, coordination and communication challenges, political and jurisdictional conflicts, and institutional and legal barriers all complicate the effective governance of cross-boundary GI (Madureira et al. 2011; Kark et al. 2015; Fernández-Pablos et al. 2021; Verdú-Vázquez et al. 2021).

Although GI connectivity within and across urban boundaries is an important GI principle, and is increasingly represented within the academic literature, further incorporation of this theme within urban GI and planning practice is needed to enhance the achievement of multifunctionality outcomes. It is therefore encouraging that methods for visualising and analysing GI connectivity to identify the spatial distribution and functional patterns of GI networks and landscapes are well represented with the academic literature. This review has identified that these methods can be placed into four main categories including spatial analysis and mapping (Meerow and Newell 2017; Tran et al. 2020; Chen et al. 2022; Lebrasseur 2022a, b; Korkou et al. 2023), remote sensing (Wang and Banzhaf 2017a, b), ecological modeling (Estreguil et al. 2019; Sun et al. 2021), and network analysis (Graviola et al. 2022; Kong et al. 2010; LaPoint et al. 2015; Zhang et al. 2019b). Rolf et al., (2018) suggested that approaches to connectivity analysis can be further refined by incorporating more precise and specialized metrics, that address ecological, social, and abiotic connectivity requirements.

Visualisation and analysis methods like these have the potential to inform the development of regional GI planning strategies to encourage structural connectivity and in turn multifunctionality outcomes at larger spatial scales. Regional scale GI studies are emerging within the academic literature (Díaz-Varela et al. 2021; Fernández-Pablos et al. 2021; Isola et al. 2022; Hou et al. 2023), focused on topics including the creation of new source patches, the conservation of important blue corridors, and establishing conservation and restoration priorities for corridors (Hou et al. 2023). Similarly, Estreguil et al., (2016) stated that enhancing GI connectivity involves firstly promoting ecological and green corridors within urban and rural areas and the transition zones between them, and secondly designing corridor networks that connect relevant ecosystems at the regional scale.

To advance this agenda and address evident research gaps, studies are needed to gather evidence on the extent to which strategies for enhancing GI connectivity, structurally and functionally, promote multifunctionality in practice. Outcome focused research is needed to support the conceptual and processes-based GI connectivity studies found within the existing literature. Here, extending evidence gathering beyond ES linked to biodiversity and species, and exploring those related to themes including health and well-being and climate change adaptation, would also be valuable. Additionally, further research into themes including planning processes to progress GI connectivity within and across urban boundaries, and on recognising and overcoming barriers to implementing cross-boundary GI connectivity strategies in practice, would be useful next steps.

### *Exploring existing literature linking GI size and GI multifunctionality*

Size is recognised as a trait with the potential to influence urban GI multifunctionality outcomes (Valente et al. 2020). Consequently, the size of urban GI sites is identified as an important factor to consider when assessing associated ES (Kabisch et al. 2016; Beichler et al. 2017; Brzoska and Spage 2020). This systematic literature review has established that GI multifunctionality research often concentrates on exploring multiple, particularly large, GI sites (see Fig. 7). It is also evident that particular attention has been paid to the capacity of different sizes of urban green spaces to sustain biodiversity and the delivery of associated ES (Lepczyk et al. 2017). Although not focused explicitly on urban areas, Fahrig (2013) found that larger habitat patches typically exhibit high levels of biodiversity (in comparison to smaller patches), which is due to factors including their ability to support sensitive species. Further research is needed to explore others forms of ES that large urban GI sites have the potential to offer, beyond those connected to biodiversity.

Although large GI sites are particularly valuable, which has been demonstrated from a biodiversity perspective, incorporating additional large GI sites within dense urban settlements will often be an unrealistic prospect. Consequently, in urban areas characterised by densely built up landscapes and high population densities, often with limited and fragmented green spaces, it is important to maximise the multifunctionality of small GI sites. It is therefore notable that research into the multifunctionality of small GI sites is much less common than research focused on large GI sites (see Fig. 7), with a particular gap in the literature regarding research into single small GI sites and their ability to generate multifunctional ES outcomes. This can be partially explained by concerns over the robustness, rigour and transferability of research findings on single small GI sites (although nevertheless research into multiple small GI sites also remains relatively rare). This gap should be addressed to develop a better understanding of the multifunctionality of small GI sites, particularly when viewed strategically as part of a wider network of small sites, to support urban GI practitioners engaged in their planning, design and implementation. Indeed, the failure to integrate small GI sites into land use and GI planning risks overlooking their localized benefits, particularly where communities existing that have limited access to larger urban green spaces.

Promising work is emerging on this topic, with studies from Europe, UK and China identifying that the implementation of small-scale GI measures offers considerable potential to address socio-environmental issues (Li et al. 2020; Back and Collins 2022), with small size pocket parks providing significant cooling and thermal comfort functions (Zhou et al. 2025). However, there are barriers to implementing small GI sites linked to their perceived value, issues with generating community buy-in, and funding and resources limitations. Small GI projects often struggle to secure funding due to their perceived lower visibility or priority compared to large-scale projects. Policymakers and practitioners may also undervalue the cumulative benefits of small GI sites, dismissing them as insignificant compared to larger green spaces. Further, the relatively high cost of small GI instillations such as green roofs can act as a barrier to their implementation in practice (Kim and Song 2019). Additional research into their potential to deliver multifunctional outcomes could therefore help to build support for small-scale GI, and assist in generating business cases for related investment.

Tran et al. (2020) evaluated two approaches for situating urban GI in Philadelphia, biodiversity-complementation (i.e., making existing GI larger and increasing patch size) and biodiversity-equity (i.e., increasing connectivity by placing GI in areas that lack GI). They found that connectivity and patch size are significant predictors of urban biodiversity with optimum habitat quality, size and connectivity metrics varying depending on the species considered. This highlights that appropriate patterns of GI connectivity and GI size characteristics are therefore not generic, but contextual. It is also apparent that the GI traits of size and connectivity must be considered in an integrated manner, not in isolation, and it is therefore encouraging to observe that GI size and connectivity are integrated within certain studies evaluating ecological functions including biodiversity conservation (Lepczyk et al. 2017; Vasiljević et al. 2018; Tran et al. 2020; Vega and Küffer 2021; Li et al. 2022). To progress related activity in practice, work is needed to better understand how to optimise GI connectivity and size traits via integrated approaches and in a way that is appropriate for locally significant species assemblages, whilst also responding to overarching contextual themes such as present day and future projected climate variables, hydrological processes and soil profiles. It will also be important to balance such biophysical considerations with the need to offer cultural ES via GI strategies in urban setting. However, this review identifies that urban GI research on these topics is lacking, and there is limited empirical data available for urban GI planners to answer questions such as how systematic strategies that manipulate GI connectivity and patch size influence ES and disservices outcomes. Interdisciplinary and transdisciplinary collaborative engagement approaches will be important to advance this agenda.

### *Acknowledging and responding to the threat of EDS*

This review has established that EDS received significantly less attention than ES within academic publications covering GI multifunctionality themes. It is also apparent that EDS that are identified within the academic literature generally focus on people and communities, which aligns with the conceptualisation of ecosystem services as requiring human beneficiaries. Our review is notable for the identification of ‘green gentrification’ as the most common disservice linked to urban GI within the academic literature. This refers to issues linked to social inequality and social exclusion that can arise when property values increase in neighbourhoods that receive GI investment. Significantly, ‘green gentrification’ was not identified by Guo et al. (2022) within their review of EDS, although this issue has been highlighted by other researchers (e.g. Curran and Hamilton 2012; Wolch et al. 2014; Haase et al. 2017; Dushkova and Haase 2020; Fisher et al. 2021; Mullenbach et al. 2021; Huang 2023).

It is also apparent that negative impacts on human health stemming from urban GI, arising from exposure to dangerous or poisonous animals for example, is a prevalent disservice theme within the literature. However, related publications generally highlight health risks without providing empirical evidence on diseases, pathogens and their vectors. This points towards a related research gap to be addressed. By identifying the prominence of anthropocentric themes such as green gentrification and health risks within the literature, this review consequently indicates a lack of an ecological conceptualisation of EDS within the academic literature, although limited reference is made to topics including detrimental impacts on biodiversity conservation (e.g. linked to habitat homogenisation and habitat competition).

Although studies have categorised EDS linked to urban GI, in some cases identifying the lack of attention paid to this topic in comparison to ES (e.g. Guo et al. 2022), our review expands on this work by considering disservices in the context of GI forms and GI connectivity and size traits, and mapping the relationships between these themes. Interventions such as enhancing connectivity between GI patches, and conserving, enhancing and potentially expanding large urban GI sites, clearly have the potential to increase urban GI multifunctionality. However, it is also important to recognise that such actions may also generate trade-offs linked to the occurrence and intensification of EDS. For example, increasing GI connectivity and size may increase pollen allergies and facilitate an expansion of urban pests (e.g. ticks, and brown rats) and disease vectors (e.g. deer), therefore increasing the risk of transmission of infectious diseases to humans (e.g. Lyme disease and typhus) (Lõhmus and Balbus 2015; Cariñanos et al. 2017). More specifically, potential risks include those posed by exposure to microorganisms include infectious disease causing agents linked to severe acute respiratory syndrome, Ebola, and vector-borne diseases like malaria, dengue fever and Lyme disease. Additionally, there are risks associated with allergens and biogenic volatile organic compounds, including increased susceptibility to allergies, isoprene emissions, and the subsequent formation of ozone, particularly during extreme heat events (Yu et al. 2024).

At the site scale, there are trade-offs between certain spatially sensitive ES provided by GI including microclimate regulation, biodiversity conservation and human well-being (Dennis and James 2017). Controlling for site area, correlational analyses revealed that these trade-offs were largely influenced by spatial configuration (including connectivity) and specific site characteristic (including the size of GI sites) (ibid). Urban planners should be encouraged to consider such trade-offs at the early stages of GI planning and decision making, taking note of themes including green space equity and spatial justice (He et al. 2023). This could be achieved by employing life cycle assessment, return-on-investment analysis, examining stakeholders’ perceptions and multi-criteria assessment (Lõhmus and Balbus 2015; Choi et al. 2021; Roman et al. 2021). This would help to moderate the risk that the emergence of EDS becomes a barrier to approaches targeted at enhancing urban GI multifunctionality. Failure to recognise, plan for and act on this issue could result in increases in certain EDS (e.g. linked to diseases and invasive species), which may act to undermine the benefits associated with GI. The results of this systematic review can inform evidence-based approaches to urban GI planning, design and management, enabling decision makers to consider trade-offs between ES and EDS to balance the pursuit of multifunctionality with the minimisation of disservices. To advance this agenda, further research is needed to explore strategies for identifying and addressing spatially driven trade-offs between ES provision and the occurrence of EDS, incorporating themes including balancing the benefits of GI for humans and nature in dense urban areas.

### *Limitations of this research*

The research findings reported in this paper emerged from the application of an established and robust systematic literature review methodology and provide a strong foundation for the proposal of insights and recommendations linked to urban GI research, planning and management. However, focusing solely on English-language academic literature due to resource constraints and language barriers introduces certain limitations. This approach may have excluded valuable grey literature (e.g. government and non-governmental reports), and such sources could be a target for further research to explore GI multifunctionality themes within governance frameworks, incorporating consideration of GI and land use planning policies and strategies produced at different spatial scales. Further, relevant non-English language regional studies may have been missed, which could potentially include case studies, innovative practices, and community-led initiatives underrepresented in academic research.

This study has demonstrated that planning literature, which was the key disciplinary focus of this review, often focuses on themes including spatial configurations, connectivity, and policy frameworks. Therefore, research linked to ecological processes, biodiversity, social equity and health impacts was underrepresented. Additionally, studies focused on interdisciplinary approaches and emerging technologies like remote sensing and machine learning may be overlooked. Further research on these underrepresented themes would be valuable. In addition, the dominance of European studies in the GI literature introduces limitations concerning the transferability of the findings. There is a risk that knowledge emerging from this literature may not adequately account for the socio-political, ecological, and cultural contexts of other global regions, leading to an overemphasis on issues and solutions that may be less applicable or feasible in different parts of the world. European urban areas often have different ecological and climatic contexts, patterns of urbanisation, policy framework, cultural perceptions of nature and urban space, and historical land-use practices than other global regions. To broaden the applicability and transferability of GI research in this field, future studies could develop and compare a more geographically diverse range of case studies, considering the variety of challenges and opportunities facing different regions, whilst also engaging more deeply with local knowledge and governance systems.

## Conclusion

Fenner (2017) stated that multiple benefits can arise coincidentally rather than intentionally through GI planning and design. To progress this agenda, more strategic and systematic approaches to planning for and capturing multiple benefits from urban GI are needed. This study emphasises the importance of paying greater attention to the contribution that manipulating GI connectivity and size traits can make to achieving multifunctional outcomes. There is a need for spatially oriented GI plans, informed by a multi-scalar and network-based understanding of existing urban GI resources, that identify targeted opportunities for GI conservation, enhancement and expansion to progress multifunctionality goals. Here, GI and land use planners must account for local contextual factors, and locally agreed objectives for urban GI identified through stakeholder collaboration and engagement, when designing strategies to maximise GI multifunctionality. This study also emphasises that the cumulative impact of small GI sites should be recognised by GI and land use planners, particularly in dense urban areas. These sites have the potential to enhance GI connectivity in such areas and deliver localised ES particularly in areas with limited access to larger green spaces. At a larger scale, policymakers should consider fostering collaboration across administrative boundaries to create landscape scale GI networks that enhance connectivity, whilst also developing policies that encourage partnerships between municipalities and stakeholders to address related governance challenge.

This research also emphasises that although enhancing connectivity and paying attention to GI size characteristics can enhance urban GI multifunctionality, there is a danger that EDS may also increase as a result. It is therefore crucial that GI planners and designers are supported in understanding disservices that can arise from urban GI, and how negative trade-offs can be accounted for and potentially lessened or avoided. Here, the development of risk assessments targeted at considering ES and EDS (and associated trade-offs) that may emerge from planned or proposed GI activities would be useful to explore. To support such activity, further research is needed into the ecological and socio-economic processes that generate EDS in urban settings, in addition to approaches that can be implemented via urban GI planning and design in response. More specifically, it is necessary to explore how different GI traits (e.g., size, connectivity) influence the balance between ES and EDS under various biophysical and socio-economic conditions. This would support urban planners and GI practitioners working to realise multiple ES through manipulating GI connectivity and size traits, whilst also minimising the risk that EDS could arise as a result of such activities.

## Supplementary Information

Below is the link to the electronic supplementary material.

Supplementary file1 (DOCX 216 kb)

## Data Availability

No datasets were generated or analysed during the current study.
