# Supplementary material for: Exploring the relationship between urban green infrastructure connectivity, size and multifunctionality: a systematic review
Source: Landsc Ecol. 2025 Mar 10;40(3):61. doi: 10.1007/s10980-025-02069-1 (PMC11893650; doi:10.1007/s10980-025-02069-1)

**Appendices**

Appendix 1. Coding categories and descriptions

| **Coding category** | **Coding category description** | **Coding question(s)** | - **Coding options** |
| --- | --- | --- | --- |
| Case studies | The case studies used in the paper (if included). | Does the paper include a single case study or multiple case studies? | - Single case study, Multiple case studies (more than 1 case). |
| Country(ies) of origin | Geographical location(s) of the study by country (may be more than one). | What is the geographical location of the study (focusing on continents and countries)? | Continents: Asia, Europe, North America, South America, Middle America, Oceania, Global.  Specific country names were added as subcodes to their corresponding continents.  *Note: If no specific geographical information is included, code as ‘global’*. |
| Methodology | Methodology employed (may be more than one). | What is the methodological foundation of the study? | Review, empirical, modelling, conceptual. |
| Study scales | Study scales of the paper (may be more than one). | What scale is the study focused at? | Multiple scales, regional, river catchment/basin, city, district, neighbourhood, site, building, not identifiable/applicable. |
| GI forms | The forms of GI included in the study (may be more than one). | What forms of GI were included in the study? | - GI (considered as a general concept without specific GI forms identified) - Greenspace (considered as a general concept without specific GI forms identified) - Park/garden - Urban agriculture/farms/allotments - Natural/semi-natural green space - Rain gardens/ rainwater harvesting - Bioswales - Permeable pavements - Greenways, alleys, boulevards - Green roofs - Green/living walls, green façades - Trees - Urban tree canopy/urban forests/woodlands - Blue infrastructure - Natural/semi-natural networks of landscapes or habitats |
| GI Size | Size as a GI trait. | Is UGI size considered? If so, what is the GI size of the study? | Not identifiable/applicableSingle small GIMultiple small GISingle large GIMultiple large GI |
| Connectivity | Connectivity (structurally) as a GI trait. | Is UGI connectivity considered? If so:  Is connectivity considered within urban areas or across urban-peri-rural boundaries?  Is connectivity between forms of infrastructure considered? | Connectivity within/across urban boundaries:Connectivity within urban areas.Connectivity across urban/peri-urban/rural boundaries.Connectivity within/between infrastructure forms:Connectivity between different GI forms.Connectivity between GI and grey infrastructure. |
| Multifunctionality | The ecosystem services (ES) and disservices that GI could/does provide. | What ES/disservices were explored? | - Did the paper address GI multifunctionality? (If it simply restates the benefits of multifunctionality, then exclude.) If yes, then: - What approach was taken to study multifunctionality?   - Empirical/Practical   - Modelling   - Conceptual - What ES and disservices were explored in the context of GI multifunctionality? (Appendix 3 used for coding) |

Appendix 2. Detailed descriptions and definitions of codes

| **Coding category** | **Coding category description** | **Coding question(s)** | **Coding descriptions** |
| --- | --- | --- | --- |
| Unique article id | Unique identification code associated with the article | The unique code generated for each article should be recorded | Number |
| Publication details | Full citation details (Authors, Title, Journal, Publication year) | Record full citation details of the paper. | Please follow Vancouver. See: https://subjects.library.manchester.ac.uk/referencing/referencing-vancouver |
| Publication type | Type of publication: journal article, report, conference paper, book chapter, other. | What is the type of publication? | Journal article, review, report, book chapter, book, conference paper, other, uncategorisable. |
| Case studies | The case studies used in the paper (if they have) | Does the paper have the single case study or multiple case studies? | - Single case study - Multiple case studies (more than 1 case) |
| Country(ies) of origin | Geographical location(s) of the study by country. May be more than one. | What is the geographical location of the study (i.e. continents, countries and cities)? | Continents: Asia, Europe, North America, South America, Oceania, Middle America, global  Note: If no specific location, code as ‘global’. |
| Methodology | Methodology employed (May be more than one) | What is the methodology used in the study? | \| Codes of methodology \| Definitions/descriptions \| \| --- \| --- \| \| Review \| “A methodological review is a type of systematic secondary research (i.e., research synthesis) which focuses on summarising the state-of-the-art methodological practices of research in a substantive field or topic." (<https://unimelb.libguides.com/whichreview/methodologicalreview>).  It also includes document/policy review here. \| \| Conceptual \| Conceptual research does not include any viable tests and experimentation, but involves the development of ideas, theories and concepts. (<https://conductscience.com/conceptual-research-vs-empirical-research/>) \| \| Empirical \| Empirical research involves research based on observation, experiments and verifiable evidence. Empirical research is based on observed and established phenomena and determines information from real involvement instead of hypothesis or conviction. (<https://www.emeraldgrouppublishing.com/how-to/research-methods/conduct-empirical-research>). \| \| Modelling \| Modelling concerns the use of models (e.g. physical, mathematical, behavioral, logical or spatial representation of a system, entity, phenomenon, or process) as a basis for simulations to develop data utilised for managerial or technical decision making. Here, this term applies quantitative analysis, and does not include conceptual models. (adapted from: <https://en.wikipedia.org/wiki/Modeling_and_simulation>) \| |
| Study scales | Study scales of the paper (maybe more than one) | What scale is the study undertaken at? | Multiple scales, regional, river catchment/basin, city, district, neighbourhood, site, building, not identifiable/applicable   \| Codes of study scales \| Definitions/descriptions \| \| --- \| --- \| \| Multiple scales \| More than one study scale. \| \| Regional \| The study is focused on regional scale.  An area is between the size of a city and a nation (Cowan, 2005, p. 324) \| \| River catchment/basin \| The study is focused on river catchments or basins.  “Catchment is the area of land drained by a watercourse.” (Cowan, 2005, p. 54) \| \| City \| The studies that collected or analysed data at a city level will be considered as the ‘city scale’.  “The aggregate of two or more towns, specialized districts and connective transportation and open space corridors.” (Cowan, 2005, p. 61) \| \| District \| The study focused on the district scale, where a district is an administrative division of a city that managed by the local government.  “Frey (1999) has suggested that an urban district might be formed of four or five neighbourhoods, with a total population of 25000-35000 and that ideally four or five districts should form a town.” (Cowan, 2005, p. 112) \| \| Neighbourhood \| The study focused at the neighbourhood scale, which refers to a geographically localized community within a city, town, or suburb area, and may consist of a single street and the buildings lining it.  “*A New Urbanist Lexicon* (McLaughlin, 2000) defines a neighbourhood as an urbanised area having a diverse range of building types, throughfares and public open spaces accommodating a variety of human activity.  *A planned neighbourhood,* Duany (2000) states, ‘is defined by an area generally circumscribed by a quarter-mile radius, which is the equivalent of a five-minute walk’.” as cited in (Cowan, 2005, p. 259). \| \| Site \| The study focused on the site scale. Sites are bigger than building scale and smaller than neighbourhood scale, and refer to locations such as parks or gardens.  “An area of land with a defined boundary” (Cowan, 2005, p. 352) \| \| Building \| Building scale relates to studies focused on interventions sited on buildings.  “A building is defined by the Royal Institute of British Architects for the purposes of its design awards schemes as ‘any structure, whether new, restored, rehabilitated or converted, which includes an elements of executed design work." (Cowan, 2005, p. 42) \| \| Not identifiable/applicable \| No scale is identifiable and/or applicable. \| |
| GI forms | The forms of GI that study included (maybe more than one) | What forms of GI were included in the study? | Specific GI forms as below (adapted from USEPA - (United States Environmental Protection Agency, 2023) and (Cowan, 2005)   \| Codes of GI forms \| Definitions/descriptions with examples \| \| --- \| --- \| \| Green infrastructure (GI) \| GI is used as a general term, without specific GI forms being identified. \| \| Green space \| Green space is used as a general term, without specific GI forms being identified. \| \| Park/garden \| “Park is a place where activities of a particular of a particular kind are concentrated, usually at a low density and served by large car parks. Examples include business park, retail park, theme park and leisure park”. (Cowan, 2005, p. 280) \| \| Urban agriculture/farms/allotments \| Urban land, either public or private, used for cultivating and distributing food, crops or medicines (adapted from: <https://en.wikipedia.org/wiki/Allotment_(gardening)>; https://en.wikipedia.org/wiki/Urban_agriculture; Wijesinghe and Thorn, 2021)  “Allotment is a garden plot in an area of similar plots. Each plot is rented by an individual, usually form the local authority.” (Cowan, 2005, p. 6) \| \| Natural/semi-natural green space \| Land covered by a mixture of land cover types, including scrub, shrubs, and grassland etc. This category excludes studies focused specifically on trees and urban forests/woodlands. \| \| Rain gardens/Rainwater harvesting system \| Rain gardens, also known as bioretention cells, are small, shallow, sunken green areas that collect and attenuate rainwater runoff from roofs, streets, and pavements.  Rainwater harvesting system measures are designed to collect and absorb runoff from streets, pavements, and car parks. The variety of systems range from the backyard rain barrel and the commercial building cistern to ground level pits. \| \| Bioswales \| Bioswales, often found along curbs and in parking lots, use vegetation or mulch to slow and filter storm rainwater flows. \| \| Permeable pavements \| Permeable pavements infiltrate, treat, and/or store rainwater where it falls. They can be made of pervious concrete, porous asphalt, or permeable interlocking paving stones. \| \| Greenways, alleys, boulevards \| “A network of spaces providing a route through an urban area for people (on foot and bicycles) and bicycles.” (Cowan, 2005, p. 168) \| \| Green roofs \| Green roofs are covered with growing media and vegetation and that enable rainfall infiltration and evapotranspiration of stored water, amongst other multifunctional benefits. \| \| Green/living walls, green façades \| They are vertical green systems that are attached to the exterior or interior of a building and including green walls system, living walls, plater green walls, vertical mobile gardens, green façades. Some green facades are designed to be mobile and used as temporary walls to create wind, solar irradiance and sound bafﬂes, some have decorative and aesthetic values. (adapted from: <https://en.wikipedia.org/wiki/Green_wall>) \| \| Trees \| Trees here means a component of nature, and an important element of GI, such as This encompasses tree planting in the streets or gardens, and heritage trees. It may refer to specific species of trees. \| \| Urban tree canopy/forests/woodlands \| Urban tree canopy/forests/woodlands refer to a collection of urban trees, that grow within city or suburb. In a wider sense, it includes any kind of woody plant vegetation growing in and around human settlements. The difference compared to the It differs from the ‘trees’ code, of ‘trees’ is this code means and refers to the layer of tree leaves, branches, and trunks stems of trees that cover the ground when viewed from above. (adapted from: <https://en.wikipedia.org/wiki/Urban_forest>) \| \| Blue infrastructure \| Blue infrastructure relates to urban water bodies infrastructure, including wetlands, ponds, lakes, streams, rivers and storm water provision. Sustainable drainage systems (SuDS) are included under this GI from. \| \| Natural/semi-natural networks of landscapes or habitats \| This refers to connected landscape networks of land, water and geological features which have been naturally colonised by plants and animals and which are accessible on foot to people. They include eco-corridors and greenbelts (East Suffolk Council, 2015). \| |
| GI Size | Size as a GI trait | Is UGI size considered? If so, what is the GI size of the study? | \| Code of GI size \| Definitions/descriptions \| \| --- \| --- \| \| Single small GI \| Individual small GI such as a green roof, green wall, bioswale, rainwater harvesting system or tree. \| \| Single large GI \| Individual large GI such as a wetland, park/garden or urban forest/woodland. \| \| Multiple small GI \| More than one small GI. \| \| Multiple large GI \| More than one large GI, which may be connected (e.g. greenways, eco-corridors, urban forests). \| \| Not identifiable /applicable \| The GI size is not identifiable or applicable \| |
| Connectivity | Connectivity as a GI trait | Is UGI connectivity considered?  Is it within the urban areas or across urban-peri-rural boundaries?  What are the forms of infrastructure? | Connectivity within/across urban boundaries:  \| Code of boundary \| Definitions/descriptions \| \| --- \| --- \| \| Connectivity within urban areas \| The study of GI connectivity within urban areas \| \| Connectivity across urban/peri-urban/rural boundaries \| The study of GI connectivity between urban and peri-urban areas, or urban and rural areas. \|  Connectivity within/between infrastructure forms:  \| Code of infrastructure forms \| Definitions/descriptions \| \| --- \| --- \| \| Connectivity between different GI forms \| The study of connectivity between forms of GI (either same form or different forms), \| \| Connectivity between GI and grey infrastructure \| The study of connectivity between GI and grey infrastructure (e.g. roads, man-made recreational facilities, residential/commercial buildings, dams/reservoirs) \| |
| Multifunctionality | GI multifunctionality approaches and ES focus | Was GI multifunctionality explored? If so, what approach was taken and which ES were considered? | Did the paper address GI multifunctionality? (If it simply restates the benefits of multifunctionality, then exclude.) If yes, then   - What approach was taken to study multifunctionality?   - Empirical/Practical: GI multifunctionality analysed/evaluated based on observations, experiments and verifiable evidence (e.g. obtained via interviews, surveys).   - Modelling: modelling GI multifunctionality using mathematical or spatial models etc.   - Conceptual: conceptualisation of GI multifunctionality in a theoretical or abstract way. - What Ecosystem services/disservices were explored within the study of GI multifunctionality (using table Appendix 3 below to code)?   - EDS   - Provisioning ES   - Regulating ES   - Cultural ES |

Appendix 3. Coding for ES classes and types following the ﻿Common International Classification of Ecosystem Services (CICES V5.1) and EDS.

| **ES section** | **CICES Class** | **Class types and descriptions** |
| --- | --- | --- |
| Provisioning | Cultivated crops | Food supply |
|  | Surface and ground water for drinking | Surface and ground water for drinking |
|  | Surface water for non-drinking purposes | Surface water used as a material (non-drinking purposes) |
|  | Trees (e.g. woodlands) | Wood and timber supply |
|  | Medicinal materials | Medicine supply |
|  | Biomass | Biomass used for energy production or as a [renewable energy](https://en.wikipedia.org/wiki/Renewable_energy) source (e.g. biofuel, solid fuel), usually produced through [agriculture](https://en.wikipedia.org/wiki/Agriculture), [forestry](https://en.wikipedia.org/wiki/Forestry) or [aquaculture](https://en.wikipedia.org/wiki/Aquaculture). |
| Regulating | Filtration/sequestration/storge/accumulation by ecosystems | - Soil quality (decomposition and fixing processes and their effect on soil quality) - Water purification - Air purification - Carbon sequestration |
|  | Micro and regional climate regulation | Urban temperature regulation (i.e., mitigating urban heat island effect, urban heat waves) |
|  | Mediation of smell/noise/visual impacts | Noise mitigation |
|  | Hydrological cycle and water flow maintenance (flood control) | Water flow regulation and runoff attenuation (including flood control) |
|  | Pollination/dispersal/conservation | - Pollination - Disease and pest control - Biodiversity conservation |
|  | Waste/wastewater recycling and reuse | Waste or wastewater recycling and reuse |
|  | Fire protection | Preventing the spread of forest/woodland ﬁres. |
| Cultural | Physical use of landscapes in different environmental settings | - Human wellbeing (i.e., physical and mental health promotion, quality of life improvement) - Aesthetic values - Social relations and recreation (i.e., leisure, entertainment, socialising activities) - Crime and traffic accident reduction - Economic value (i.e., increased business and job opportunities, increased property/market value, increased business revenue, cost reduction compared to traditional engineering solutions) |
|  | Educational and research | Educational value for citizens and children, research value |
|  | Heritage and cultural values | Heritage, cultural, religious and spiritual values, sense of place |
| ES disservices | Unpleasant odour | Odour from rotting organic matter |
|  | View blockage by plants | View blockage by plants (e.g. trees standing close to buildings) |
|  | Waste dumping | ﻿Open defecation/dumping of garbage and/or discharge of sewage/effluents into ecosystems |
|  | Space for anti-social activities/nuisances | ﻿Consumption of alcohol/drugs etc. in groups of people, security threats for women |
|  | Health problems | Presence of dangerous or poisonous animals (including diseases, pathogens and their vectors), breeding grounds for disease-spreading mosquitoes, human-tick-deer interaction and associated Lyme disease transmission risk |
|  |  | Allergies (e.g. ﻿wind-pollinated plants causing allergic reactions) |
|  |  | Accidents (e.g. linked to water, aging of vegetation, biomass fixation in roots) |
|  |  | Fear and stress (e.g. dense vegetation development) |
|  | Pest species | Alien invasive species that are harmful to local ecosystems |
|  | Habitat competition with humans | Habitat competition with humans (e.g. abundance of insects and rats) |
|  | Air quality problems | Emission of volatile organic compounds |
|  | Noise problems | Noise generated at GI sites or during their construction |
|  | Conflicts (with neighbours) | Conflicts with neighbours due to gardening issues |
|  | Homogenisation of habitat composition | Biodiversity loss, agricultural diversity loss |
|  | Green gentrification | ﻿Gentrification and further marginalization and displacement of disadvantaged communities; uneven distribution of or inaccessibility to green spaces caused green gentrification and social inequality |

Appendix 4: Heatmap of the relationship between each type of ecosystem (dis)services that GI provided, and associated GI forms, based on numbers of reviewed publications (n.b. the darker colours (both red and green) reflect higher numbers of publications)


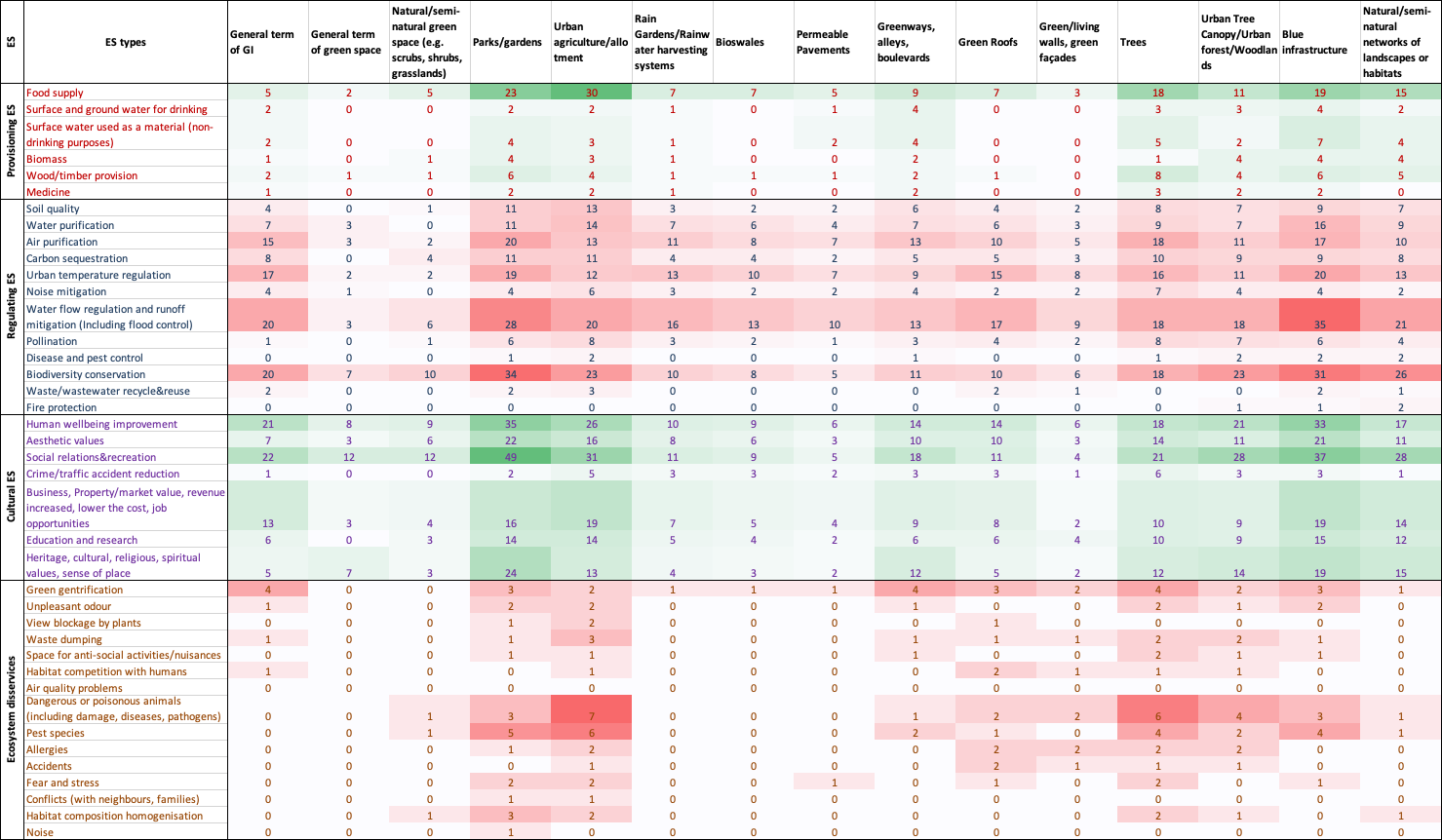

Supplement: Supplementary file 1 — Supplementary file1 (DOCX 216 kb) [file 10980_2025_2069_MOESM1_ESM.docx]
